# A Discrete Transition Zone Organizes the Topological and Regulatory Autonomy of the Adjacent *Tfap2c* and *Bmp7* Genes

**DOI:** 10.1371/journal.pgen.1004897

**Published:** 2015-01-08

**Authors:** Taro Tsujimura, Felix A. Klein, Katja Langenfeld, Juliane Glaser, Wolfgang Huber, François Spitz

**Affiliations:** 1 Developmental Biology Unit, European Molecular Biology Laboratory, Heidelberg, Germany; 2 Genome Biology Unit, European Molecular Biology Laboratory, Heidelberg, Germany; Medical Research Council Human Genetics Unit, United Kingdom

## Abstract

Despite the well-documented role of remote enhancers in controlling developmental gene expression, the mechanisms that allocate enhancers to genes are poorly characterized. Here, we investigate the *cis-*regulatory organization of the locus containing the *Tfap2c* and *Bmp7* genes *in vivo*, using a series of engineered chromosomal rearrangements. While these genes lie adjacent to one another, we demonstrate that they are independently regulated by distinct sets of enhancers, which in turn define non-overlapping regulatory domains. Chromosome conformation capture experiments reveal a corresponding partition of the locus in two distinct structural entities, demarcated by a discrete transition zone. The impact of engineered chromosomal rearrangements on the topology of the locus and the resultant gene expression changes indicate that this transition zone functionally organizes the structural partition of the locus, thereby defining enhancer-target gene allocation. This partition is, however, not absolute: we show that it allows competing interactions across it that may be non-productive for the competing gene, but modulate expression of the competed one. Altogether, these data highlight the prime role of the topological organization of the genome in long-distance regulation of gene expression.

## Author Summary

The specificity of enhancer-gene interactions is fundamental to the execution of gene regulatory programs underpinning embryonic development and cell differentiation. However, our understanding of the mechanisms conferring specificity to enhancers and target gene interactions is limited. In this study, we characterize the *cis*-regulatory organization of a large genomic locus consisting of two developmental genes, *Tfap2c* and *Bmp7*. We show that this locus is structurally partitioned into two distinct domains by the constitutive action of a discrete transition zone located between the two genes. This separation restricts selectively the functional action of enhancers to the genes present within the same domain. Interestingly, the effects of this region as a boundary are relative, as it allows some competing interactions to take place across domains. We show that these interactions modulate the functional output of a brain enhancer on its primary target gene resulting in the spatial restriction of its expression domain. These results support a functional link between topological chromatin domains and allocation of enhancers to genes. They further show that a precise adjustment of chromatin interaction levels fine-tunes gene regulation by long-range enhancers.

## Introduction

Differential regulation of gene expression transforms shared genomic information into the cell type-specific programs underlying organismal development and homeostasis. In vertebrates, it is not uncommon to find gene regulatory elements, in particular enhancers, hundreds of kilobases away from their target gene (reviewed in [1], [2]). The mere scale of this genomic distance raises the question of how enhancers and promoters can find each other, and how enhancers distinguish between their specific target and other neighboring genes, which may even lie much closer. Understanding the molecular basis of such specific interactions is essential as their impairment can lead to mis-expression of the normal target gene [3], [4] or to inappropriate activation of neighboring genes [5]–[8], with often severe phenotypic consequences [7], [9]–[12].

Enhancers can typically activate transcription from different promoters, a property that is part of their initial definition [13] and which has been amply used to assess enhancer activity. Many enhancers act pervasively across their endogenous genomic surroundings [14], [15], and enhancer sharing is not unusual between neighboring genes, particularly within multigenic clusters [16]–[22]. Noteworthy, this can also occur between genes with no functional relationship except genomic proximity [9], [23]–[25]. Nonetheless, in many loci, adjacent genes exhibit distinct expression patterns, implying the existence of mechanisms that limit the promiscuous potential of enhancers.

Different mechanisms and genomic elements have been invoked to explain enhancer-target gene specificity. They can be divided in two main categories, depending on whether they may promote interactions (eg. nature of the promoter, tethering elements [26], [27]), or block them. Amongst the latter, insulators prevent contact of an enhancer with an adjacent promoter, when placed in between [28]–[30]. This capacity of insulators to organize the genome in separate regulatory compartments designate them as critical components in ensuring specificity of *cis-*regulatory interactions [31]. However, only a handful of insulator elements have been functionally assessed in their native genomic context, and therefore their mode(s) of action is still poorly understood. Contrary to earlier models, a growing body of evidence suggests that insulators do not function autonomously, but rather through higher-order 3D conformations [32].

The necessity to consider the genome's three-dimensional organization is further highlighted by genome-wide high-resolution interaction maps obtained by chromosomal conformation capture techniques [33]. These studies revealed that the genome is compartmentalized in topologically-associating domains (TADs) [34], [35]. TADs have been proposed to contribute to gene expression by limiting enhancer action [36], [37]. In support of this view, genes located within the same TAD tend to be expressed coordinately [35], [38], and TADs have been found to encompass the regulatory domains defined by long-range enhancer activities [15], [39]. Recent works have addressed the finer-scale structural organization of TADs, revealing a complex hierarchy of interactions, which may contribute to mediate long-distance interactions between enhancers and promoters [40], [41] and to subdivide them into distinct regulatory domains [15]. In most instances, the functionality of structural contacts is difficult to evaluate precisely and the causal relationship between structural conformation and gene regulation remains unclear.

To better understand the relationships between 3D structural properties of the genome and enhance-promoter allocations, we focused on a large interval of approximately 0.5 Mb containing two different developmental genes, *Bmp7* and *Tfap2c*. These two genes, which encode a secreted signaling molecule and a nuclear transcription factor, respectively, are active in multiple tissues and organs during embryogenesis [42]–[48]. Both genes have promoter architectures compatible with tissue-specific and long-distance regulatory inputs [49]. Their expression overlaps in the limbs, forebrain and branchial arches of mid-gestation mouse embryos, while in other contexts, their expression is specific of one or the other and exclusive. Therefore this locus constitutes an ideal system to study the control of long-distance enhancer specificities.

To investigate the regulatory organization of this locus, we used a transposon/recombination-based chromosomal engineering approach [14]. We show here that the genomic interval consists of two largely independent regulatory domains, corresponding to each of the two genes. Analysis of the chromatin conformation of re-engineered genomic configurations identified a central transition zone (TZ) that defines different topological sub-domains. Importantly, the allocation of enhancers to one or the other gene is determined by this partition. Altogether, our data support the view that the topological organization of the genome restricts enhancers to specific domains, determining therefore their “specific” target gene choice. Interestingly, we found that the presence of *Bmp7* in *cis* has a mild influence on the expression level of *Tfap2c* in the developing forebrain, indicating that the position of the two genes to different topological domains does not lead to an absolute insulation.

## Results

### Mapping the regulatory landscapes of the *Tfap2c-Bmp7* locus

**Figure 1:**
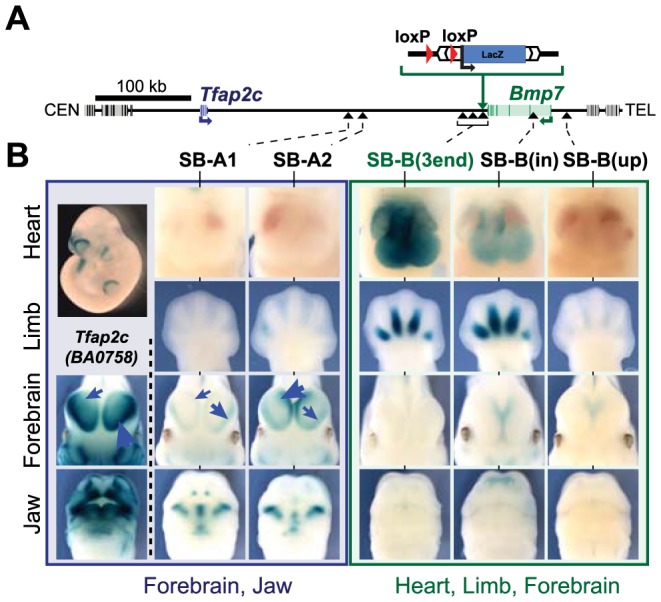
The *Tfap2c-Bmp7* locus consists of two regulatory domains. (A) A schematic representation of the *Tfap2c-Bmp7* locus, including the position of the SBlac insertions. Gene bodies are depicted as grey boxes with darker bars representing their exons. *Tfap2c* and *Bmp7* are blue and green, respectively. The centromeric (CEN) and telomeric (TEL) ends of the chromosome are to the left and right of the diagram, respectively. The *Sleeping Beauty* transposon carrying a *loxP* site and *LacZ* reporter was first targeted into the immediate downstream region of *Bmp7* along with an additional *loxP* sequence. Integration sites obtained upon remobilisation of the transposon are indicated by black arrowheads. (B) LacZ staining patterns of the transposon lines in the heart, limb, forebrain and the jaw as well as the staining of the BA0758 gene trap line in the forebrain and jaw are shown. Limbs: E12.5 embryos; other tissues: E11.5 embryos. Note that the intensity of the LacZ staining in the lateral and medial parts of the forebrain varies among BA0758, SB-A1 and SB-A2, as indicated by the blue arrows. Additional stages and views available in S1 Fig.

To determine the regulatory organization of the *Tfap2c-Bmp7* locus, we adapted the GROMIT (Genome Regulatory Organization Mapping with Integrated Transposons) strategy [14]. Firstly, at the 3′ end of the endogenous *Bmp7* gene, we inserted a transgene consisting of a *Sleeping Beauty* transposon comprising 1) a regulatory sensor gene (a *LacZ* reporter under the control of a short naïve synthetic promoter region derived from the human *β-globin* gene [14], [50]) and 2) a *loxP* site. After establishment of a mouse line carrying the correct insertion, we removed the selection marker used to identify candidate targeted ES clones, a step which left behind an additional *loxP* site, next to the *Sleeping Beauty* transposon. We designated this allele as SB-B(3end) (Fig. 1). By serial remobilisation of the transposon *in vivo* [14], we obtained several insertions located in this region of mouse chromosome 2 (S1 Table). Of these, seven insertions were distributed along the *Tfap2c-Bmp7* locus (Fig. 1A): three very close to SB-B(3end) (within 23 kb), one (SB-B(up)) 20 kb upstream of *Bmp7*, and another one in the first intron of *Bmp7* (SB-B(in)). The remaining two (SB-A1 and SB-A2) lie within the large intergenic region separating *Tfap2c* and *Bmp7*. In parallel, we established a mouse line (BA0758) from an ES clone carrying a *βgeo* gene-trap insertion in *Tfap2c* [51].

### The *Tfap2c*-*Bmp7* locus consists of two distinct regulatory domains

We analyzed the expression pattern of the regulatory sensor at different insertion sites in E10.5 to E12.5 mouse embryos, at stages when *Tfap2c* and *Bmp7* show both shared and specific expression patterns (Fig. 1, S1 Fig.). The two insertions located between *Tfap2c* and *Bmp7* (SB-A1 and -A2) showed very similar LacZ staining in the oro-facial region, the branchial arches, and in the forebrain (Fig. 1B, left). These three expression domains are strikingly consistent with reported expression patterns of *Tfap2c* [42] and particularly with the *Tfap2c LacZ* gene-trap allele (Fig. 1B- S1 Fig.). This overlap and agreement in expression suggested that SB-A1 and -A2 were included in the *Tfap2c* “regulatory domain” [15]. The expression of the reporters showed however different relative intensity between the lateral and medial part of the forebrain: while BA0758 and SB-A1 were preferably expressed in the lateral forebrain, with weaker expression in the medial region, SB-A2 showed the inverse pattern, with a stronger medial than lateral LacZ staining. Such position-effects (the promoter is the same for SB-A2 and SB-A2) are not uncommon within regulatory domains [15], [52]. They may reflect the presence in the locus of several forebrain enhancers with distinct medial/lateral activity and different range of action.

These forebrain expression domains were not observed with any of the four insertions located within the 23-kb region at the 3′end of *Bmp7* (Fig. 1B, S1 Fig.), suggesting that the telomeric limit of *Tfap2c* regulatory domain is upstream of this region. More distant insertions in *Bmp7* (SB-B(in); SB-B(up)) showed weak medial-only forebrain expression at E11.5, with no lateral expression detected, as also observed for *Bmp7* [44]. None of the six telomeric insertions showed the characteristic oro-facial expression observed with the *Tfap2c*-associated insertions. In contrast, they shared several common expression domains not reported by the SB-A1 and –A2 insertions (Fig. 1B). The four insertions at the 3′end of *Bmp7* and SB-B(in) showed all prominent staining for *LacZ* expression in the developing heart (from E10.5 to E12.5), and in the interdigital mesenchyme (at E12.5). SB-B(up) displayed only faint LacZ staining in the interdigital mesenchyme, and no staining in the heart. However, LacZ expression from this position overlapped characteristically with other SB-B insertions in the whiskers, nasal pits, and forebrain (S1 Fig.), defining collectively a regulatory domain distinct from the one associated with *Tfap2c*. This domain includes *Bmp7*, and accordingly, several of the reported activities overlap with known *Bmp7* expression domains [47], [53]. Some regions of the *Bmp7* expression domain were not reflected accurately in the activity of the SB reporters, being either missing or spatially expanded. These differences may arise from the limited range of action of some promoter-proximal enhancers [53], and/or from the different post-transcriptional stability and dynamics of LacZ compared to the endogenous *Bmp7* transcripts.

Overall, the regulatory activities detected by the sensor differed significantly between the centromeric and telomeric part of the locus, and highlighted two distinct and non-overlapping regulatory domains, each defined by multiple distinct tissue-specific activities, one domain corresponding to *Tfap2c* and the other to *Bmp7*. We focused for subsequent analyses on the forebrain (medial and lateral) and heart, as representative markers of these two domains. In these two tissues, the expression pattern of the different genes is stable from E10.5 and E12, contrasting with the dynamic expression of these genes in the developing limbs and face. Also, for these two expression domains, it is technically possible to dissect from embryos the part where the gene or the enhancer is active, without the contribution of too many non-expressing cells.

### Enhancers in the intergenic region control either *Bmp7* or *Tfap2c*

**Figure 2:**
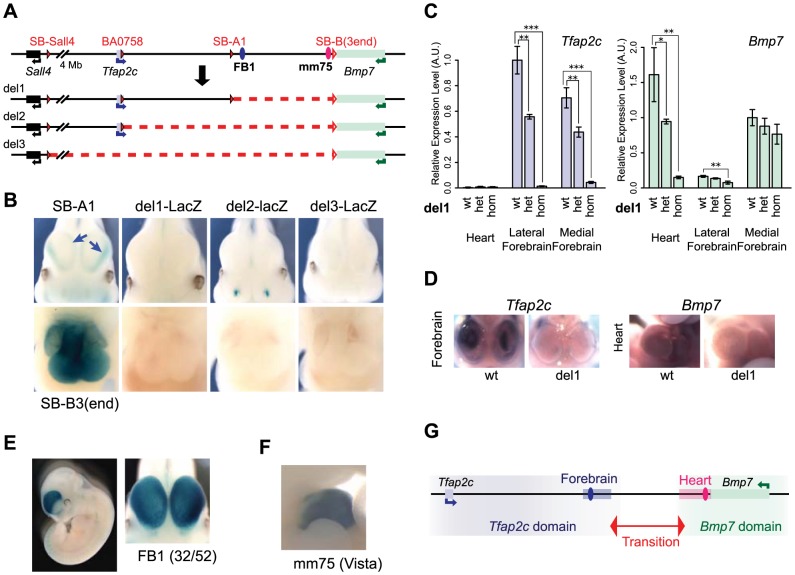
Deletion alleles localise enhancers. (A) A schematic representation of the deletions generated. The *loxP* sites used for CRE-mediated recombination are depicted as filled red triangles for the one carried with the transposon. Open triangles indicate both the position of the static *loxP* at the 3′end of *Bmp7* and the position of the *LacZ* reporter gene in deletions produced by TAMERE. (B) LacZ staining patterns of the three deletion lines in forebrain (top) and heart (bottom) in E11.5 embryos, in comparison to SB-A1 and SB-B3(3end), respectively. The deletions led to complete loss of the both lateral and medial expression in the forebrain (blue arrows in the SB-A1 embryo). (C) Relative expression levels of the *Tfap2c* and *Bmp7* mRNAs in the heart, lateral and medial forebrains from E11.5 embryos measured by RT-qPCR in del1 homozygous, heterozygous, and control (wt) genotypes. For each gene, expression levels were normalized with *Gapdh*. Expression of the wild type allele in the lateral forebrain for *Tfap2c* and in the medial forebrain for *Bmp7*, respectively, was set as 1. The error bars represent s.d. from three biological replicates. Statistical significance was assessed by a two-sided *t*-test. *p<0.05; **p<0.01; ***p<0.001. (D) *In situ* hybridization of the wild type and del1 embryos at E10.5 with the anti-sense RNA probes for *Tfap2c* and *Bmp7*. (E) Enhancer activity of FB1 on the *LacZ* reporter gene. 32 out of 52 transgenic embryos showed broad forebrain expression. (F) The mm75 element drives specific expression in the mouse embryos at E11.5 (from VISTA enhancer browser: http://enhancer.lbl.gov). (G). Regulatory domains along the *Tfap2c*-*Bmp7* interval. The forebrain enhancer (FB1) and the heart enhancer (mm75) are depicted with blue and pink ovals. A light blue (resp. pink) rectangle represents the region encompassing the H3K27ac peaks present in the segment deleted in del1, in the forebrain (resp. heart) (S3 Fig.).

To further characterize the functional relevance of these two domains and associated enhancers, we used *in vivo Cre-*mediated recombination to engineer chromosomal deletions removing either the telomeric half or the whole of the intergenic region (Fig. 2). Each deletion was produced using a combination of *loxP* sites in *cis and trans* [54] in order to keep the *LacZ* sensor at the deletion breakpoint (see Materials and Methods). With the TAMERE strategy, we also obtained a large duplication, reciprocal to del3 (S2 Fig.). All three deletions led to a complete loss of *LacZ* expression in the embryonic heart and forebrain (Fig. 2B) suggesting that the enhancers detected by SB-A1 and SB-B(3end) lie in the region encompassed by del1. Dup3-lacZ embryos showed LacZ expression in the heart similar to SB-B(3end), corroborating the presence of the heart enhancer(s) at the 3′ side of *Bmp7* (S2 Fig.). These deletions also provided information on the locations of additional enhancers associated with other expression domains (S2 Fig.).

We next determined if the enhancers present in the del1 interval contributed to *Tfap2c* and *Bmp7* expression by whole-mount *in situ* hybridization and RT-qPCR (Fig. 2C–D). In del1 homozygous embryos, *Bmp7* expression was drastically reduced in the heart compared to wild-type littermates, while the very weak expression of *Tfap2c* in the heart was unaffected (Fig. 2C). In the forebrain, where both genes are expressed, we found an almost complete loss of *Tfap2c* expression in both the medial and lateral parts of del1 embryos. In contrast, *Bmp7* expression was barely affected and showed only a slight reduction in the lateral forebrain (Fig. 2C).

These analyses demonstrated a critical role of elements located within the del1 segment for the specific expression of *Tfap2c* in the forebrain and of *Bmp7* in the heart, respectively. Several peaks enriched for chromatin marks associated with active enhancers (H3K27ac, EP300) have been detected within this region in the forebrain and the heart of E11.5 embryos [55]–[57] (S3 Fig.). Interestingly, the distribution of these regions is coincident with the location of the two regulatory domains. Many forebrain H3K27ac peaks are located between *Tfap2c* and SB-A1/A2, while the only ones present around *Bmp7* lie in the first intron of the gene. Conversely, heart H3K27ac-enriched elements cluster around the 3′ end of *Bmp7*. H3K27ac peaks were also identified outside of the del1 region around the locus. The forebrain H3K27ac peak adjacent to *Bmp7* could account for its unaffected expression in del1; however, the role of the predicted forebrain and heart enhancers located respectively centromeric and telomeric to del1, respectively, remained unclear, as they were seemingly unable to confer significant activity to the reporter gene or to the endogenous genes in these tissues, in the absence of del1 sequences.

To confirm that del1 contained enhancers with the expected activities, we cloned FB1, an evolutionarily conserved element enriched for both H3K27ac and EP300 in the forebrain, upstream of the regulatory sensor construct. In this transgenic assay, FB1 drove specific and reproducible LacZ expression in the forebrain in E11–12 embryos (Fig. 2E), including the *Tfap2c* expression domain. However, FB1 appeared broadly and equally active in both medial and lateral forebrain, contrasting with the restricted expression detected by the same reporter gene than the one used in the transgenic assay when inserted in the endogenous locus on either side of FB1. In this context, it showed alternatively preferential expression in the lateral (SB-A1, like *Tfap2c*) or medial (SB-A2). These differences suggested that additional factors – possibly the other H3K27ac-region present in the vicinity (see below) – may modulate FB1 intrinsic activity in a position-dependent manner. Amongst the predicted heart enhancers, one of them (mm75) had been tested previously [58] and reported to have broad enhancer activity in the heart of E11.5 mouse embryos (Fig. 2F).

Taken together, these data demonstrated that the del1 region contained heart-specific and forebrain-specific regulatory element(s) critical for the expression of *Bmp7* in the heart, and of *Tfap2c* in the forebrain, respectively. Importantly, these elements appeared to be dispensable for the regulation of one another's genes. These selective influences and the separate location of the different enhancers further confirmed the partition of this genomic interval into two distinct regulatory domains containing enhancers which act exclusively on one or the other gene (Fig. 2G).

### Topological organisation of the locus

**Figure 3:**
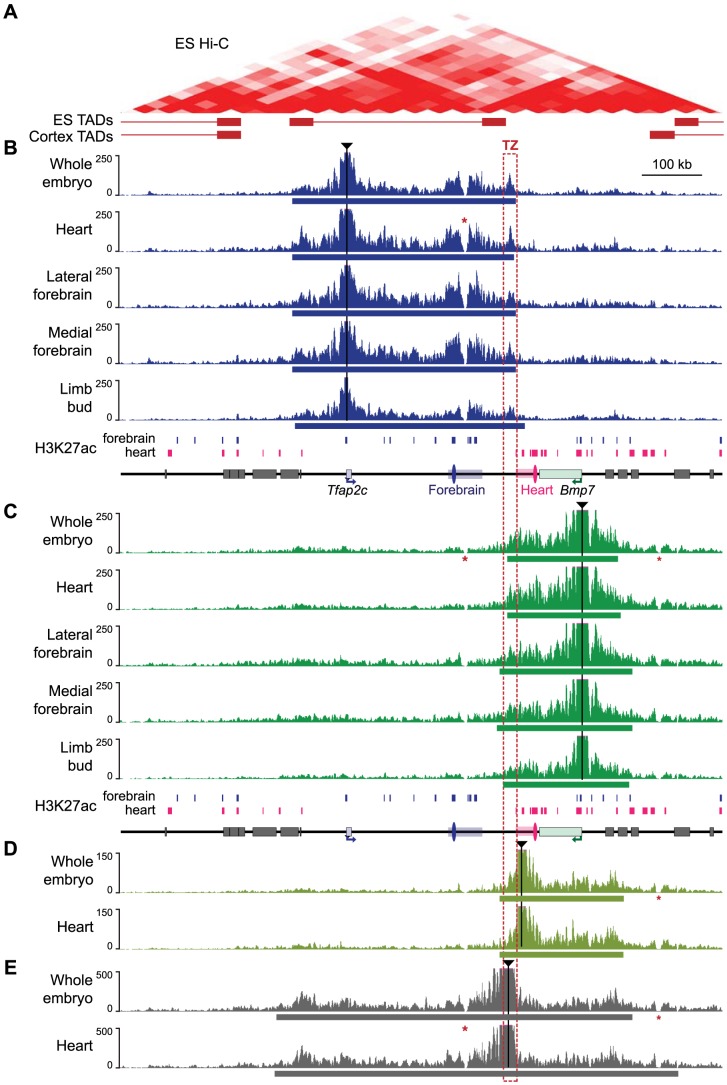
4C profiles describing the conformational structure of the *Tfap2c-Bmp7* locus. (A) Hi-C heat-map of the *Tfap2c*-*Bmp7* locus in mouse ES cells (top) and corresponding TADs identified in ES cells and adult cortex (bottom; shown by whiskered red bars) (data from Dixon et al. 2012; aligned with the other panels). (B–E) 4C-contact profiles for the different viewpoints (indicated by black triangles): promoter of *Tfap2c* (B) and *Bmp7* (C), adjacent to the TZ (D) and within the TZ (E). Five different tissues (whole embryos, heart, lateral forebrain, medial forebrain at stage E11.5, limb buds at E12.5) were examined with the two promoter viewpoints (B, C). Only whole embryos and heart samples were used for the additional viewpoints (D, E). The estimated primary interaction domains are indicated by a bar below the corresponding 4C plots. The region of overlap of the different primary interaction domains (TZ) is outlined with a dashed red box. Stars (*) indicate two regions with low mappability [84] accounting for the absence of signal over these positions. For comparison, a schematic representation of the region including H3K27ac peaks detected in forebrain and heart chromatin (S3 Fig.) is shown below panels B and C. Surrounding gene bodies are represented with grey boxes. The FB1 and mm75 enhancers are shown as blue and pink ovals, respectively. Associated rectangles indicate the extended enhancer regions encompassing the additional H3K27ac regions detected within the del1 segment.

We next investigated how the regulatory subdivision of the locus corresponded to its topological organization. Hi-C data available for mouse ES cells and cortex [34] suggests that the locus has a relatively loose topological structure, confined between two prominent topologically associating domains (Fig. 3A, S4 Fig.). To determine the pattern of physical contacts involving *Tfap2c* and *Bmp7*, we carried out circular chromatin conformation capture experiments followed by high-throughput sequencing (4C-Seq) using the promoters of these two genes as viewpoints (Fig. 3). We performed these 4C-Seq analyses on dissected samples where one and/or the other gene were expressed (E11.5 heart, medial and lateral forebrain) and whole body of E11.5 embryos (where most cells are non-expressing either of the two genes). We also included samples from E12.5 limbs, which comprised a majority of non-expressing cells.

For both viewpoints, the 4C profiles highlighted a large primary interaction domain characterized by high 4C read counts (Fig. 3B, C). We applied a segmentation algorithm [59] to delineate this primary domain in the different conditions (S2 Table). The calculated primary interaction domains for a given viewpoint were nearly identical across the different tissue samples. The 4C profiles were predominantly similar between samples, with the exception of a moderate increase of the 4C signals over the enhancers associated with each gene in the tissues in which they are active (for *Tfap2c*: FB1 and flanking H3K27ac-enriched regions in the brain samples; for *Bmp7* mm75 and surrounding H3K27ac-enriched regions in the heart sample). We confirmed the increased interactions of *Tfap2c* with FB1 and of *Bmp7* with mm75 in an independent 3C experiment (S5 Fig.). Importantly, the reciprocal 3C experiment with FB1 as a viewpoint showed that it contacted strongly *Tfap2c* in the forebrain, but not in the heart, and had much weaker/rarer contacts with *Bmp7*.

Noteworthy, the *Tfap2c* domain and the *Bmp7* domain end shortly before the edges of the flanking TADs detected in mouse ES cells [34], consistent with the notion that these 4C primary domains corresponded to the structural conformation adopted by the locus. In all samples, the primary contact domains of one gene included the enhancer regions we found associated with it, but excluded the ones associated with the other gene. Nevertheless, we observed a consistent overlap between the two domains, demarcating a region of about 10- to 30-kb region, which we termed the transition zone (TZ). To further characterize this region, we used two additional viewpoints for 4C analysis (Fig. 3D–E). Contacts observed from a viewpoint located just before the centromeric end of the *Bmp7* primary domain showed extensive overlap with the latter, extending broadly over *Bmp7* but not stopping almost abruptly at the TZ (Fig. 3D). Similarly, FB1 showed only weak contact with positions located on the other side of the TZ (S5C Fig.). This asymmetry in the distribution of contacts suggested the TZ indeed corresponds to a conformational transition between two different conformations. Importantly, a viewpoint located in the TZ itself showed prominent contacts extending towards both genes (Fig. 3E), consistent with the strong 4C signals observed over the TZ in the reciprocal 4C experiments.

Next, we performed 4C analyses on del1 homozygous embryos, where the TZ region was deleted together with a larger part of the locus, including the different enhancers (S6 Fig.). In this context, we observed a wide extension of the contacts made by *Tfap2c* (resp. *Bmp7*) in the telomeric (resp. centromeric) region, over distances larger than the size of the deleted region. At the same time, the centromeric (resp. telomeric) profiles remained highly similar between WT and del1. Interestingly, the intervals with frequent contacts by *Tfap2c* and *Bmp7* now largely overlapped, as if they “merged” into one domain only limited by the adjacent TADs (S6 Fig., S3 Table). These new extended contacts supported the notion that the TZ may contribute to delineate two distinct structural domains. However, as del1 also significantly reduced the linear distance between *Tfap2c* and *Bmp7*, we decided to use other types of alleles to challenge the structural and regulatory organization of the locus and to test the influence of the TZ on these.

### Chromosomal rearrangements led to enhancer re-allocation

**Figure 4:**
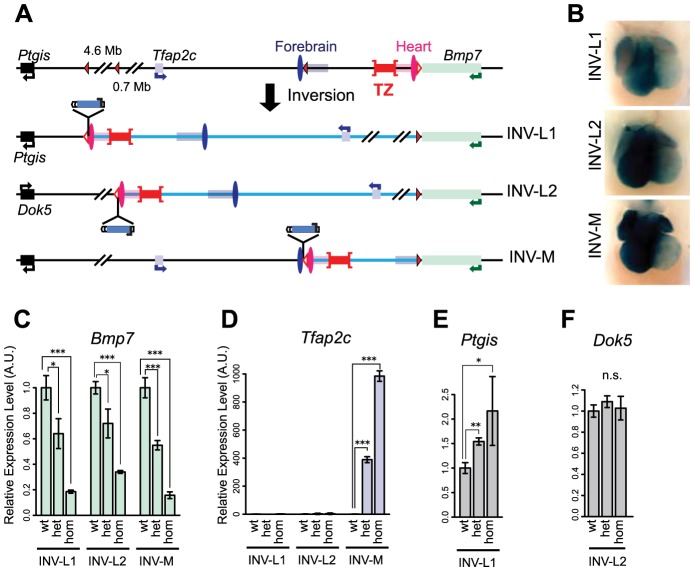
Inversion alleles reallocate the target of the heart enhancer. (A) A schematic representation of the three different inversions obtained in this study, with *loxP* sites as triangles, genes as plain boxes, and enhancers as ovals (FB1 or mm75) or grouped in rectangles for the ones predicted by chromatin marks (FB1/forebrain H3K27ac: blue, mm75/heart H3K27ac: pink). The TZ is represented by a whiskered red bar. In the generated inversion lines, the heart enhancer(s) were brought next to the LacZ reporter. (B) LacZ staining of the three inversion lines in E11.5 heart. Quantification by mRNA RT-qPCR of expression levels of *Tfap2c* (C), *Bmp7* (D), *Ptgis* (E) and *Dok5* (F) in the inversion alleles. Expression levels in wild type (wt) were normalized as 1. The error bars represent the s.d. of three biological replicates. The statistical significance was assessed by a two-sided Student's t-test. *p<0.05; **p<0.01; ***p<0.001; n.s.: non-significant.

We used insertions carrying *loxP* sites in the opposite orientation to the one left at the SB-B(3end) position *in cis* to engineer three balanced inversions by CRE-mediated recombination (Fig. 4A, S1 Table). In INV-L1 and -L2, the distance between *Bmp7* and the heart enhancer increased to 5.7 and 1.1 Mb, respectively, whereas the relative order and distances between *Tfap2c*, the enhancers and the TZ region were unchanged (S7 Fig.). In INV-M, the heart enhancer was now equidistant from *Bmp7* and *Tfap2c* (187 and 207 kb, compared to distances of 80 kb and 312 kb in the wild-type allele, with mm75 taken as reference). However, in this allele, the TZ was now located between *Bmp7* and the heart enhancer(s). With each inversion, the *LacZ* reporter remained adjacent to the heart enhancer region and displayed its normal heart expression (Fig. 4B, S7 Fig.), demonstrating that these rearrangements did not disrupt heart enhancer activity. In the three inversions, *Bmp7* expression was strongly reduced in the heart, comparable to levels observed with del1 (Fig. 4C). In contrast, *Tfap2c* expression was enhanced by a thousand-fold in the heart of INV-M animals (Fig. 4D), implying that in this genomic configuration, the heart enhancers now activated *Tfap2c* instead of *Bmp7*. This complete switch of the heart enhancer(s) from *Bmp7* to *Tfap2c* coincided with the new relative position of the TZ. The importance of the position of the TZ was further supported by a lack of up-regulation of *Tfap2c* in INV-L1 and INV-L2 (Fig. 4D), where its location with regards to the TZ/heart enhancers remained unchanged. In INV-L1, we instead found an up-regulation of *Ptgis* (Fig. 4E), which was now located on the other side of the TZ, next to mm75. As *Ptgis* was closer to the heart enhancer (S7A Fig.) we were unable in this case to fully rule out a possible influence of distance on promoter choice. However, in INV-L2, *Dok5*, the new gene juxtaposed “next to” the heart enhancer(s) opposite to TZ was much further away than *Tfap2c* (1.1 Mb versus 0.3 Mb). In this context, neither *Dok5* (Fig. 4F) nor *Tfap2c* were up-regulated in the heart, ruling out the possibility that the heart enhancer(s) act simply by default the nearest gene.

### Intrinsic asymmetric distribution of interactions around the TZ

**Figure 5:**
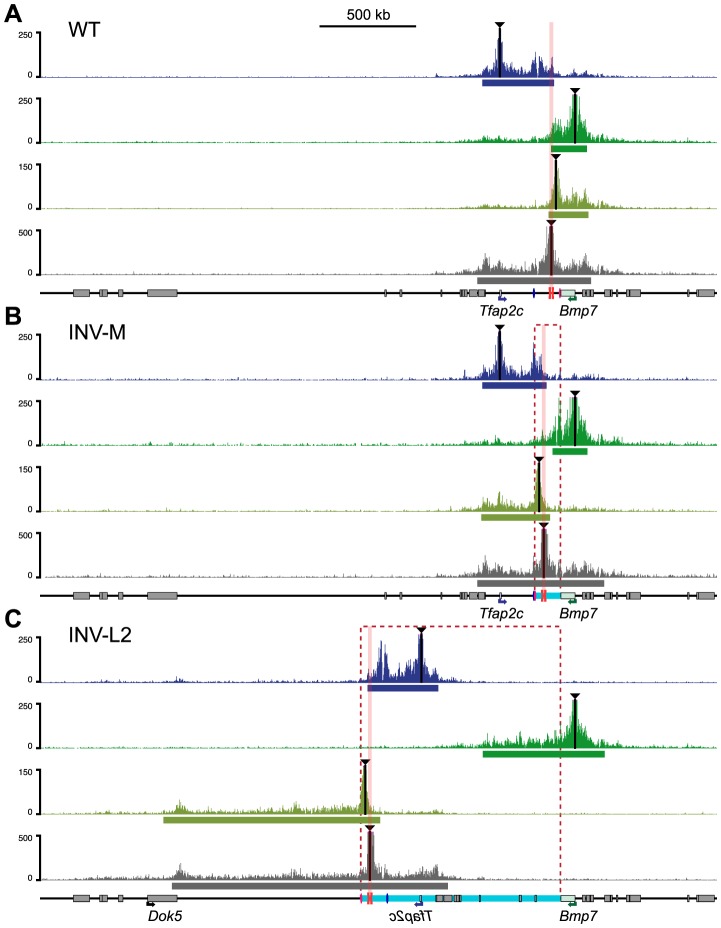
Redistribution of the interaction domains upon chromosomal inversions. 4C profiles were compared amongst WT control (A), INV-M (B) and INV-L2 (C) alleles for the four viewpoints indicated with black triangles. For inversion plots, the genomic coordinates were reordered to take the genomic rearrangements into account: hence, representated profiles correspond to the actual genomic structure of each allele. Representations of the data aligned on the reference (WT) genome are available in S8 and S9 Figs. Dashed rectangles and light-blue bars represent the regions inverted in the INV-M and INV-L2 alleles. The position of the TZ is marked by pink columns. The heart (mm75) and forebrain (FB1) enhancers are depicted as pink and blue ovals, respectively. The bars below each plot represent the corresponding primary interaction domain.

To examine at the consequences of these rearrangements on the structural conformation of the region, we performed 4C experiments on INV-M and INV-L2 embryos (Fig. 5, S8–S10 Figs.). In INV-M, as in WT controls, *Tfap2c* showed robust interactions over a domain extending up to the TZ. Due to the inversion, this domain now included the heart enhancer, which displayed much stronger interaction with *Tfap2c* than those observed in WT (S8A Fig., pink versus grey arrow), a result consistent with mm75 now activating *Tfap2c*. Conversely, the new primary interaction domain of *Bmp7* stopped at the TZ, with a very reduced 4C signal over the heart enhancer in INV-M when compared to WT (S8D Fig., grey versus pink arrow). The viewpoint located between mm75 and TZ, which was part of the *Bmp7* interaction domain in WT, showed in INV-M broad and extended contacts overlapping with the *Tfap2c* interaction domain, ending at the TZ region (Fig. 5B). Interestingly, the inversion had no effect on the 4C profile of the TZ-associated viewpoint, which extended on both sides in all configurations. Thus, in INV-M as in WT, the locus appeared structurally partitioned at the TZ: instead of maintaining their normal contacts and regulatory preferences, genes and regulatory elements established new interactions, depending on their respective position in relation to the TZ.

In INV-L2 embryos, the 4C profile of *Tfap2c* appeared generally unchanged and did not expand across the TZ into its new flanking region. The TZ-flanking viewpoint remained still limited by the TZ, but highlighted on the other side a broad domain of nearly 1 Mb in the *Dok5*-*Cbln4* gene desert, which is now adjacent to it. The 4C signal was strongly diminished before reaching the promoter of *Dok5*, which may explain the lack of up-regulation of this gene in the heart of INV-L2 embryos (Fig. 4F). Again, the TZ itself contacted both flanking regions, the relocated *Tfap2c* domain, and the new *Dok5*-*Cbln4*. Importantly, in INV-L2, *Bmp7* showed broad contacts over the region now present at its 3′end, extending for up to 0.5 Mb further in the *Cbln4* locus, supporting the notion that the presence of the TZ limited the extent of the *Bmp7* contact range (Fig. 5C, S3 Table).

Remarkably, the new distribution of 4C contacts in the different rearrangements appeared to follow quite strictly the relative position of the TZ. It did not appear to depend on the nature of the flanking sequences themselves. The directional bias of contacts made by the viewpoint flanking the TZ is the same in the different configurations (WT, INV-M and INV-L2) (S10 Fig., on the right), irrespectively of the flanking sequences.

### Fine-tuning of *Tfap2c* forebrain expression across the TZ

**Figure 6:**
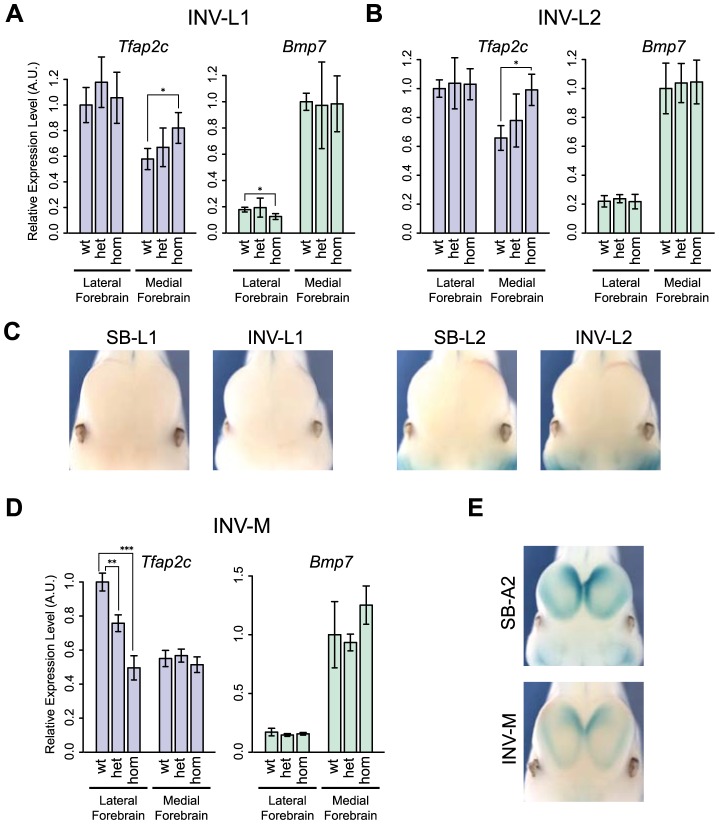
Changes of gene expression in the forebrain following genomic inversion. Quantification by RT-qPCR of the relative expression levels of the *Tfap2c* and *Bmp7* mRNAs in the lateral and medial forebrain for INV-L1 (A), INV-L2 (B) and INV-M (D), normalized as in Fig. 2. Error bars represent the s.d. of three biological replicates. The statistical significance was assessed by a two-sided Student's t-test. *p<0.05; **p<0.01; ***p<0.001. (C) Absence of LacZ staining in the forebrain of SB/INV-L1 and SB/INV-L2 E11.5 embryos. (E) LacZ staining of SB-A2 (up) and INV-M (bottom) E11.5 embryos.

The expression and structural changes observed in the heart suggested that the TZ behaved as a simple insulator region. In INV-L1 and INV-L2, the *Tfap2c* domain was fully maintained and unaffected by the genomic rearrangements. Therefore one would expect little impact on *Tfap2c*. However, we observed an up-regulation of *Tfap2c* in the medial telencephalon in both alleles (Fig. 6A–B). This up-regulation is unlikely to be caused by the juxtaposition of new forebrain enhancers, as the regulatory sensor did not detect any forebrain activity in L1 and L2 position, in either the inverted or non-inverted configurations (Fig. 6C). We noted that in INV-L1 and –L2, *Bmp7*, which is strongly expressed in the medial forebrain, was relocated away from *Tfap2c* and its forebrain enhancer. This rearrangement had no effect on *Bmp7* expression in the forebrain, suggesting that it was the presence of *Bmp7 in cis* that negatively influenced *Tfap2c*. Supporting this hypothesis, we did not observe any up-regulation of *Tfap2c* in the medial forebrain of INV-M embryos (Fig. 6D), where *Bmp7* remained adjacent to the *Tfap2c*. These observations prompted us to re-examine the 4C profiles. As stated before, the intensity of the 4C signals diminished strongly beyond the TZ region. However, we observed that the 4C contacts made by the *Bmp7* promoter, albeit weak, were stronger over the *Tfap2c* domain than over the region located symmetrically from the viewpoint (S9 Fig., green boxes). Reciprocally, *Tfap2c* showed weak but consistent interactions with the *Bmp7* region in WT and INV-M (S9 Fig., blue boxes), interactions which are not observed with a symmetrically located region, or with the region at the equivalent place in INV-L2. To further test if the INV-L1 and –L2 up-regulation of *Tfap2c* depended on the removal of *Bmp7*, we produced INV-Bmp7 which consists in a simple inversion of the gene itself. Consequently, *Bmp7* remained adjacent to the *Tfap2c* domain, and separated from it by the TZ (S11A Fig.). In this configuration, we did not observe significant changes of *Bmp7* or *Tfap2c* expression, with the exception of a small reduction of *Bmp7* expression in the lateral forebrain. Altogether, these results supported that the simple presence of an active *Bmp7* in *cis*, despite the presence of the TZ region, can affect *Tfap2c* expression in the medial forebrain.

We also noted that INV-M led to a significant reduction of *Tfap2c* expression in the lateral forebrain (Fig. 6D), even if the genomic region between *Tfap2c* and FB1 was unaffected. This reduction could result from the relocation to the other side of the TZ of two forebrain-specific H3K27ac-enriched regions included in INV-M. As we observed neither a concomitant up-regulation of *Bmp7* (Fig. 6D) nor changes in the activity reported by the sensor (Fig. 6E), it is possible that these elements may not act autonomously but rather modulate the long-range action of FB1.

## Discussion

We show here that the neighboring *Tfap2c* and *Bmp7* genes are controlled by distinct set of enhancers acting specifically on one or the other gene. Since we observed in a balanced genomic rearrangement a switch of enhancer-gene preferences, the specificity of these enhancers for one or the other gene cannot result exclusively from differences in their promoter structures, as proposed for other situations [49], [60]. In contrast, our results indicate that, for this locus, the regulatory interactions are in a large part determined by the relative position of the different elements, as reported for other complex regions [7], [61], [62].

Our 4C experiments showed that *Bmp7* and *Tfap2c* lie in genomic domains that share limited physical contacts. These domains were only weakly demarcated in the available Hi-C data in ES cells [34]. Therefore, it is unclear if the *Tfap2c* and *Bmp7* domains correspond to adjacent sub-TADs [41], or weak TADs in a rather unstructured region. However, the distinction between these different levels of spatial segregation of the genome may in part be semantic, based on arbitrary thresholds, which may not be pertinent for gene regulation. We showed here that the distinct enhancers that regulate each gene (this work, [53], [63]) reside and act within the corresponding conformational domain, further supporting the functional relevance of the structural partition we described in establishing distinct domains of regulation [15]. Furthermore, we showed that a balanced rearrangement exchanging the relative position of genes, enhancers and the TZ region led to a concomitant redistribution of physical and regulatory interactions. The switch of the heart enhancer from *Bmp7* to *Tfap2c* and the patterns of contacts observed in this configuration demonstrate together that the topological separation in two distinct domains is key to allocate distant enhancers to one or the other gene.

We observed extensive similarities in the 4C profiles between the different cell tissues assayed, irrespective of the expression state of the corresponding genes. This indicates that the *Tfap2c-Bmp7* locus adopts a rather generic conformation which undergoes limited changes in response to transcriptional activity. Such a constitutive folding has also been described for other loci [34], [40], [64]–[66]. It suggests that the structural partitioning of the locus into two domains pre-exists and guides regulatory interactions, instead of deriving from directed interactions between active genes and enhancers.

Our functional dissection of the locus highlights that the transition zone separating the two domains has an important role in organizing this topological subdivision. The fusion of the interaction profiles of the two promoters and the centromeric extension of the *Bmp7* interaction domain upon removal of the TZ strongly argue in favor of the TZ preventing interactions between *Bmp7* and *Tfap2c*. The different balanced inversions further demonstrate that the TZ organizes this topological separation irrespectively of the nature of its flanking sequences. Interestingly, the TZ region interacts robustly with both flanking regions, suggesting that the topological segregation between *Tfap2c* and *Bmp7* may arise from its action as an interaction sink or decoy, not as a blocker or repulsive element. TAD “boundaries” often displayed strong interactions with regions flanking them on both side [34], suggesting that this behavior could be a rather general feature of topological transitions. The TZ does not appear to coincide with a region of constitutive transcription, contrarily to a large subset of typical TAD boundaries [34]. It is flanked by and includes several constitutive CTCF sites [38]. CTCF sites have been proposed to anchor long-range interactions and to act, together with cohesin and Mediator complexes, as master regulators of the chromosomal 3D conformation [67], [68]. However, as only a subset of CTCF sites act as insulators [15], [69], and as depletion of CTCF only mildly impacts chromosomal topologies [70] and long-range gene regulation [71], the precise role of these sequences – and of other regions of the TZ – would need to be directly assessed.

**Figure 7:**
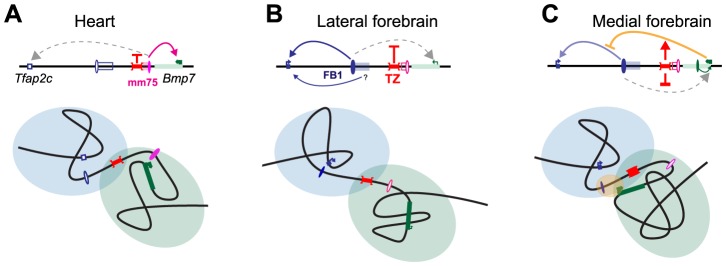
Structural partitioning controls enhancer-target gene allocation and modulates enhancers' effective activity on target genes. Genes and enhancers are shown as rectangles and ovals, respectively. Active promoters and enhancers are marked with arrows and plain colors. The TZ organizes the locus into two distinct, partially overlapping spatial conformations (represented by light blue and green circles), where genes and enhancers can interact. In the heart (A) and forebrain (B), this situation prevents action of one enhancer on a gene in the other domain. In the lateral forebrain, enhancers adjacent to FB1 may contribute to *Tfap2c* expression. In the medial forebrain (C), the active *Bmp7* promoter may compete, non productively, for the forebrain enhancer, and interferes (marked by a yellow oval) with its action on *Tfap2c*. The TZ may control the strength and therefore the consequences of this interference on *Tfap2c* expression.

With regard to the allocation of the heart enhancer, the TZ behave similarly to a classical insulator (Fig. 7). However, the analysis of INV-L1 and –L2 indicates that the TZ does not provide complete shielding from external influences, as the presence, beyond the TZ, of an active *Bmp7* promoter can interfere with the expression of *Tfap2c* in the medial forebrain. Although contacts between *Bmp7* and *Tfap2c* and its associated forebrain enhancer(s) are limited and even insufficient to lead to productive interactions (i.e. activation of *Bmp7*), they are nonetheless present at higher than background level. Our data suggests that they may be frequent and/or strong enough to perturb the regulation of *Tfap2c* by its forebrain enhancer(s), most probably through promoter competition. Several studies have reported that promoters have a tendency to come into close proximity [40], [72], [73], particularly when they are co-active and linked. Our analysis indicates that the TZ appears to counteract this generic promoter clustering by limiting admixing of the two domains, but it does not however totally prevent the diffusion of regulatory influences between them. The functional impact of these influences underscores the difficulties of defining functional thresholds for the interaction data obtained with 4C or Hi-C. It also emphasizes that topological domains should not be considered as strict autarchic units: topological separation does not exclude neighborly relationships and semipermeable borders. Transformation of the intrinsically broad forebrain activity of FB1 into the graded expression pattern shown by *Tfap2c* may involve additional neighboring enhancer elements, as hinted to by the INV-M data. However, our observations suggest that the permeability of the TZ to active *Bmp7* may also contribute to this fine-tuning (Fig. 7C). In operational terms, the TZ should be considered as a rheostatic controller rather than as a strict insulator.

Interestingly, a sequence orthologous to FB1 is present between *Tfap2c* and *Bmp7* in the coelacanth, but not in teleosts or sharks (S12 Fig.). This indicates that the origin of FB1 can be traced back to the ancestor of the lobe-finned fishes. In contrast, the sequence of the TZ region is far less conserved, suggesting a more recent origin. Expression of *Bmp7* in the forebrain is likely an ancestral feature, as it is shared amongst *Bmp7* orthologues and paralogues [44]. Conversely, *Tfap2c* is the only member of its family expressed in the forebrain [42], [45], and the only one directly adjacent to a *Bmp* gene. The evolution of FB1 as a forebrain enhancer may have been favoured by the pre-existing expression of *Bmp7* in this tissue, as suggested for other loci [74]–[76]. In this scenario, we suggest that *Bmp7* may have initially been the primary target of this emerging enhancer. The evolution of a region with insulating-like activity would have make FB1 available to *Tfap2c*. Interestingly, the forebrain expression of *Tfap2c* regulates the formation of basal progenitors in the developing cortex in mammals [77] and variations of this expression levels, in space and time, have been proposed to account for the increased number of cortical neurons present in higher primates [77]. Changes in gene expression changes are usually attributed to evolution of enhancers or promoters [78]. Our results indicate that a simple change of the filtering capacity of the TZ may also provide evolution with means of modulating gene expression.

## Materials and Methods

(See S1 Text for details)

### Generation of the different transgenic lines and chromosomal rearrangements

The initial allele used to produce SB-B(3end) was obtained by homologous recombination in ES cells (E14). The targeting construct comprised: the SB8 transposon [79]; an additional *loxP* site outside of the transposon; a neomycin resistance gene under the control of the PGK promoter that are flanked by two FRT sequences. The homology arms (chr2:172686051–172689701 and chr2:172689702–172694528 (NCBI37/mm9)) were amplified by PCR and then attached to the targeting construct above. After transformation and selection in ES cells, correctly targeted clones were injected into donor C57BL/6J blastocyst. Germline transmission was obtained from one chimera. The FRT-flanked selection cassette was then removed by breeding with hACTB-FLPe mice, leaving only the transposon and the loxP sequence outside of it at the site (allele SB-B(3end)). The ES clone BA0758 was obtained from BayGenomics, verified by PCR genotyping, and injected to establish a *Tfap2c*-gene trap line.

The SB transposon was remobilised and new insertions were mapped as described before [14]. Alleles carrying the different deletions, duplications and inversions were produced by *in vivo* genomic engineering [18], [54], using the 129S1/Sv-*Hprt^tm1(cre)Mnn^*/J CRE line [80]. Deletions del1 and del3 were obtained by recombination in *cis* between the static *loxP* site at the end of *Bmp7* and the one moved along with the transposed insertion SB-A1 and SB-Sall4, respectively. To keep the regulatory sensor at the deletion breakpoint, we also produced another version of these deletions, del1-LacZ and del3-LacZ, by CRE-mediated recombination in *trans* [54], between the *loxP* site from SB-B(3end) and the one at SB-A1 and SB-Sall4, respectively. For the del2-lacZ allele, we used a recombination in *trans*, between SB-B(3end) and BA0758. Mice were genotyped by PCR (see Supplemental Experimental Procedures).

Mouse experiments were conducted in accordance with the principles and guidelines in place at European Molecular Biology Laboratory, as defined and overseen by its Institutional Animal Care and Use Committee, in accordance with the European Convention 18/3/1986 and Directives 86/609/EEC and 2010/63/EU.

### Gene and reporter gene expression analysis

LacZ staining and whole-mount *in situ* hybridization was carried out following standard protocols. For RT-qPCR, total RNA was extracted from the frozen tissues using RNeasy kit (QIAGEN), and then cDNA was synthesized using the ProtoScript II First Strand cDNA Synthesis Kit (New England Biolabs). The quantitative PCR was performed using StepOne Real-Time PCR System with SYBR green reagent (Applied Biosystems). Gapdh was used to normalize expression level for each sample. The extra-embryonic membranes were used for PCR-genotyping of the embryos.

### *In vivo* enhancer assay

We cloned the FB1 enhancer (chr2:172551998–172555000, NCBI37/mm9) upstream of the reporter gene used in SB8, in a lentiviral vector [81]. The transgenic provirus was produced in HEK293 cells as described elsewhere [81]. Briefly, the virus was micro-injected under the zona pellucida of one-cell embryos which were maintained in culture up to the blastocyst stage. Embryos were then reimplanted into foster mothers and, at stage E11.5 or E12.5, stained for LacZ activity and genotyped.

### 3C assay, 4C library preparation, sequencing and data analysis

To prepare the 3C library we dissected out the heart and the lateral and medial forebrains from E11.5 C57BL/6 embryos. The cells were dissociated, fixed and then processed following the protocol in Splinter et al. [82]. The fixed genomic DNA was digested with *NlaIII* enzyme and subsequently self-ligated. To quantify the ligation products of interest, we conducted qPCR with TaqMan probes. qPCR was performed with four technical replicates, and for each value, mean and standard deviation were plotted.

For the 4C analyses, the 3C libraries were first prepared as described above from the respective tissues with *NlaIII* enzyme. They were then subjected to digestion by *DpnII* and ligation. After purification of the circularized DNA, inverse PCR was performed to obtain 4C libraries. Reading primers had 3–6 nucleotides of tag sequence, to allow for demultiplexing of the pooled libraries after sequencing. PCR products were purified, mixed altogether and sequenced on a HiSeq 2000 (Illumina). For data analysis, we first demultiplexed the FASTQ files of the 4C sequencing libraries and then aligned them to the mm9 reference genome using Bowtie version 1.0.0 [83]. To normalize with regard to library size, we divided the counts by the total number of counts on the viewpoint chromosome (chr2) for each library and multiplied these values by 1,000,000 (“RPM normalization”). We then smoothed the counts over adjacent fragments, using a window size of 11 fragments. Details are available in Supplementary Information. Sequencing data of the 4C libraries is deposited at ENA (Study Accession ERP005557)

## Supporting Information

S1 Fig LacZ staining of the transposons in the *Tfap2c*-*Bmp7* locus. (A) Lateral views of whole embryos stained with X-gal, from E10.5 to E12.5. The scale bar is 1 µm. Numbers at the bottom indicate the corresponding IDs in TRACER database (see S1 Table) [79] (B) Frontal view of SB-B(in) and SB-B(up) embryos at E12.5. Arrows indicate LacZ expression in the forebrain (FB), nasal process (NP) and the whiskers (W). (C) Magnified view of the LacZ expression in the mammary placodes (MP) in BA0758 and SB-B(up) embryos at E12.5. (D) LacZ expression in the limbs of BA0758, SB-A1 and SB-A2 at E10.5.
(TIF)

S2 Fig Localisation of enhancers. (A) A schematic representation of the SB-Sall4 insertion allele and rearrangements obtained from it. The pink and blue ellipses represent the heart and forebrain enhancers, respectively. The brown ellipse represents the putative position of other enhancers associated with *Sall4*. (B) LacZ staining of SB-Sall4 (top), dup3-LacZ (middle) and del3-LacZ (bottom) in E10.5 to E12.5 embryos. A pink arrow indicates LacZ expression in the heart gained in dup3 in comparison to SB-Sall4, which showed no staining there (dashed pink arrow). LacZ expression in the posterior neural tube and midbrain (indicated by brown arrows) was maintained after the duplication, but was lost with the deletion, suggesting that the corresponding enhancers are located on the telomeric side to the SB-Sall4, as indicated by the brown ellipse in (A). The green arrow indicates LacZ staining in the interdigital limb mesenchyme. (C) LacZ staining in the jaw at E11.5 (left) and in the limb at E12.5 (right) in the three deletion lines together with SB-A1 and SB-B(3end) lines. JD and JL represent the distal jaw and the lateral mandibule, respectively (purple arrows). (D) Enhancers for interdigital limb mesenchyme and jaws could be roughly located within the locus, as depicted by shaded ovals. The characteristic interdigital expression of SB-B(3end) was observed both with del1 and del3, but surprisingly not with del2. Since the centromeric breakpoint used for del3 (SB-Sall4) showed no expression in interdigital mesenchyme (B), we conclude that the corresponding limb enhancer should not be centromeric to the deleted region, and therefore it should lie telomeric to SB-B(3end), in the *Bmp7* gene region. Consistent with this, a limb enhancer was identified in the first intron of *Bmp7* [53], overlapping with prominent H3K27ac and EP300 peaks (S3 Fig.). The absence of limb expression in del2-lacZ may result from the presence next to the sensor of the *Tfap2c* promoter, which may out-compete it in this configuration, or from another type of position-effect. The expression patterns in the developing jaws observed with SB-A1 and –A2 comprise multiple sites of expression. Expression in the lateral mandible was lost with del1-LacZ, whereas expression in the distal jaw was maintained in this deletion, but absent in del2-lacZ and del3-lacZ (S2C Fig.). These observations suggested that the corresponding enhancers should be located between A1 and B(3end), and between *Tfap2c* and SB-A1, respectively.
(EPS)

S3 Fig Chromatin maps in the *Tfap2c-Bmp7* locus. Potential enhancer regions in the *Tfap2c*-*Bmp7* locus in the heart, forebrain and limbs were identified by ChIP-seq and transgenic studies. Red bars at the top row represents the transposon insertions obtained in this study. The del1 region is indicated by the horizontal red line. Critical enhancer regions for the forebrain and heart (series of H3K27ac-enriched peaks present in the del1 region) are indicated with blue and pink rectangles, respectively, with the experimentally tested FB1 and mm75 elements shown as ovals. For the three different tissues (E11.5 forebrain: blue; E11.5 heart: pink; E11.5 limb: brown), H3K27ac read-counts tracks, as well as H3K27ac and EP300 peaks are shown. Data was obtained from Gene Expression Omnibus: forebrain and heart H3K27ac (GSE52386; [55]); limb H3K27ac (GSM1371056; [85]); EP300 peaks from [56], [57], [86].
(EPS)

S4 Fig Chromatin conformation of the *Tfap2c*-*Bmp7* locus from Hi-C data. (A) Hi-C TADs around the *Tfap2c*-*Bmp7* locus (data from [34]). The *Tfap2c*-*Bmp7* locus is flanked by two well-defined TADs on both sides, but it has itself a less pronounced structural organization, despite the prediction of a weak TAD around *Tfap2c* in ES cells.
(EPS)

S5 Fig 3C-qPCR. The interaction profiles of the *Bmp7* promoter (A), *Tfap2c* promoter (B) and FB1 enhancer (C) are shown. The pink, blue and green plots are the data from the heart, the lateral forebrain and the medial forebrain, respectively.
(EPS)

S6 Fig 4C profiles of the del1 configuration in comparison to WT. The black triangles indicate the viewpoint fragments. The estimated primary interaction domains are depicted by bars below the corresponding 4C plot.
(EPS)

S7 Fig LacZ staining and RT-qPCR of INV-L1 and INV-L2. (A) A schematic representation of the SB-L1 and SB-L2 transposon insertions and the associated inversions. (B, C) Comparison of the LacZ expression patterns between SB-L1 and INV-L1 (B) and between SB-L2 and INV-L2 (C). A pink arrow indicates the heart in expressing embryos.
(EPS)

S8 Fig 4C profiles of INV-M. (A-D) 4C profiles of the INV-M configuration compared with WT plotted against the reference WT genomic coordinates. The black triangles indicate the viewpoints. The arrowheads indicate the position of the heart enhancer in A and D. Note that this region gained an interaction with the *Tfap2c* promoter upon inversion (pink arrowhead), while losing it with the *Bmp*7 promoter. (B, C) Percentages of normalized interaction read counts (excluding the 10-kb region around the viewpoints) for the inverted region and the flanking 400-kb regions on the both sides.
(EPS)

S9 Fig An extended view of the 4C profiles. Black triangles indicate the viewpoints. Whole-embryo samples from WT, INV-L2, INV-M and del1 configurations, as well as samples from WT lateral and medial forebrain, are shown as indicated by the labels to the left. The dashed red rectangles indicate the inverted or deleted regions of the respective alleles (deleted regions are put in yellow). Dark blue and green squares highlight higher 4C interaction between *Tfap2c* and *Bmp7*, compared to regions located at the same distance (squared with light blue and green), either on the opposite side or on the same side but in a rearranged allele. 4C profiles were normalized and plotted against the reference genomic coordinates.
(EPS)

S10 Fig Intrinsically asymmetric distribution of interactions around the TZ. Plots show cumulative normalized 4C reads from the viewpoint (black arrowheads) located between the TZ and the heart enhancer (ellipse) up to the physical distance indicated on the x-axis for the left and right sides, separately. 4C reads mapped within the 10-kb distance from the viewpoint were excluded. Purple and blue are 4C data of WT heart and whole embryo, respectively. Red and green are data of INV-M and INV-L2 4Cs, respectively. WT and inverted configurations of the locus are represented below the chart. The viewpoint and the TZ are fixed in this plot for all the configurations, so the left is always the TZ side and the right is the other direction. INV-L1 configuration is also shown in the same manner, illustrating that *Ptgis*, which was upregulated in the heart following inversion, is now at the right side together with the heart enhancer.
(EPS)

S11 Fig An intra-domain inversion of *Bmp7* did not impact on the expression levels of either *Tfap2c* or *Bmp7* in the medial forebrain. (A) A schematic representation of INV-Bmp7. (B, C) LacZ expression pattern of INV-Bmp7, in E11.5 and E12.5 embryos (B). Close-up views of E11.5 heart and forebrain (C). (D) Quantification of mRNA expression levels for *Tfap2c* (left panel) and *Bmp7* (right) in the inversion allele. Expression levels of the wild type allele in the lateral forebrain for *Tfap2c*, and in the medial forebrain for Bmp7, were normalised as 1, respectively. Error bars represent the s.d. of three biological replicates. Statistical significance was scored by a two-sided Student's t-test between the wild type and the mutation. *p<0.05; **p<0.01; ***p<0.001. ns: non-significant.
(EPS)

S12 Fig Synteny conservation of the *Tfap2c*-*Bmp7* locus. (A) *Tfap2c* and *Bmp7* genes are adjacent in genomes from mammals to lobe-finned fishes, but not in ray-finned fish or cartilaginous fish genomes. The FB1 enhancer is also conserved in coelacanth, but not in the teleost lineage (blue ellipse). (B) Paralogous genes of *Tfap2c* and *Bmp7* in mice are not located next to each other, even though they are often on the same chromosome. Arrows indicate the direction of transcription of the genes indicated by the boxes.
(EPS)

S1 Table List of transposon insertions and associated rearrangements. IDs in the TRACER database [79] corresponding to the line names used in this study are indicated in the left column. The column of “Parental Line” indicates from which transposon line the respective insertions were obtained.
(DOCX)

S2 Table Estimated coordinates of the primary interaction domains. Coordinates are on chromosome 2, using the NCBI37/mm9 assembly.
(DOCX)

S3 Table Estimated coordinates of the primary interaction domains in rearranged alleles. Coordinates are on chromosome 2, using the NCBI37/mm9 assembly, with those within inverted regions reordered accordingly.
(DOCX)

S1 Text Supplemental Materials and Methods.
(DOCX)
