# Supplementary figures and images for: A Discrete Transition Zone Organizes the Topological and Regulatory Autonomy of the Adjacent Tfap2c and Bmp7 Genes
Source: PLoS Genet. 2015 Jan 8;11(1):e1004897. doi: 10.1371/journal.pgen.1004897 (PMC4288730; doi:10.1371/journal.pgen.1004897)

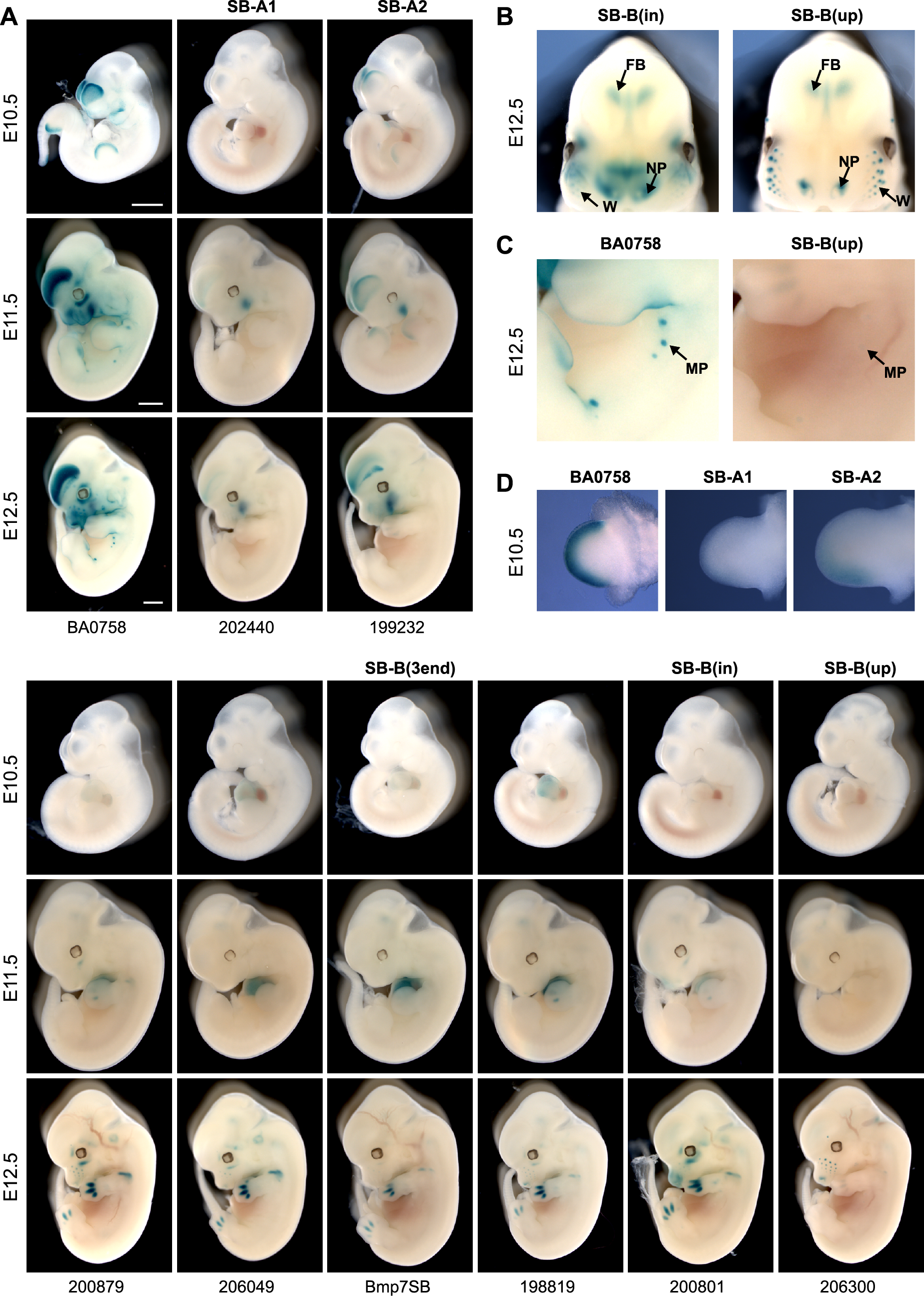

Supplement: S1 Fig — LacZ staining of the transposons in the Tfap2c-Bmp7 locus. (A) Lateral views of whole embryos stained with X-gal, from E10.5 to E12.5. The scale bar is 1 µm. Numbers at the bottom indicate the corresponding IDs in TRACER database (see S1 Table) [79] (B) Frontal view of SB-B(in) and SB-B(up) embryos at E12.5. Arrows indicate LacZ expression in the forebrain (FB), nasal process (NP) and the whiskers (W). (C) Magnified view of the LacZ expression in the mammary placodes (MP) in BA0758 and SB-B(up) embryos at E12.5. (D) LacZ expression in the limbs of BA0758, SB-A1 and SB-A2 at E10.5. (TIF) [file pgen.1004897.s001.tif]
