# Supplementary material for: A Discrete Transition Zone Organizes the Topological and Regulatory Autonomy of the Adjacent Tfap2c and Bmp7 Genes
Source: PLoS Genet. 2015 Jan 8;11(1):e1004897. doi: 10.1371/journal.pgen.1004897 (PMC4288730; doi:10.1371/journal.pgen.1004897)
Supplement: S1 Table — List of transposon insertions and associated rearrangements. IDs in the TRACER database [79] corresponding to the line names used in this study are indicated in the left column. The column of “Parental Line” indicates from which transposon line the respective insertions were obtained. (DOCX) [file pgen.1004897.s013.docx]

| **TRACER ID** | **Short Name** | **Chr.** | **Position (mm9)** | **LoxP Orientation** | **Rearrangements made from the insertion** | **Parental Line** |
| --- | --- | --- | --- | --- | --- | --- |
| 205312 | SB-L1 | 2 | 167096172 | minus | INV-L1 | Bmp7SB |
| 206063 | SB-Sall4 | 2 | 168617845 | plus | del3, del3-LacZ, dup3-lacZ | 198819 |
| 205918 | SB-L2 | 2 | 171656649 | minus | INV-L2 | Bmp7SB |
| 202440 | SB-A1 | 2 | 172540246 | plus | del1, del1-LacZ | Bmp7SB |
| 199232 | SB-A2 | 2 | 172556092 | minus | INV-M | Bmp7SB |
| 200879 |  | 2 | 172667410 | minus |  | Bmp7SB |
| 206049 |  | 2 | 172678863 | minus |  | 198819 |
| Bmp7SB | SB-B(3end) | 2 | 172689701 | plus |  | NA (targeted) |
| 198819 |  | 2 | 172690678 | minus |  | Bmp7SB |
| 200801 | SB-B(in) | 2 | 172745981 | minus |  | Bmp7SB |
| 206300 | SB-B(up) | 2 | 172783163 | minus | INV-Bmp7 | Bmp7SB |

**Table S1.** **List of transposon insertions and associated rearrangements.**

For each line used in this study, the corresponding IDs in the TRACER database [79] are indicated in the left column. The column of “Parental Line” indicates from which line the respective insertions were obtained by *in vivo* transposition [14].
