# Supplementary material for: A Discrete Transition Zone Organizes the Topological and Regulatory Autonomy of the Adjacent Tfap2c and Bmp7 Genes
Source: PLoS Genet. 2015 Jan 8;11(1):e1004897. doi: 10.1371/journal.pgen.1004897 (PMC4288730; doi:10.1371/journal.pgen.1004897)
Supplement: S2 Table — Estimated coordinates of the primary interaction domains. Coordinates are on chromosome 2, using the NCBI37/mm9 assembly. (DOCX) [file pgen.1004897.s014.docx]

| **Viewpoint/Tissue** | **Coordinates of boundaries (lower-upper estimates)** |
| --- | --- |
| Tfap2c/Whole embryo | Centromeric: 172286221 (172285831-172287052)  Telomeric: 172657199 (172656822-172657592) |
| Tfap2c/Heart | Centromeric: 172286221 (172285831-172287052)  Telomeric: 172654155 (172653500-172655105) |
| Tfap2c/Lateral forebrain | Centromeric: 172286221 (172285831-172287052)  Telomeric: 172657199 (172656822-172657592) |
| Tfap2c/Medial forebrain | Centromeric: 172286221 (172285831-172287052)  Telomeric: 172657029 (172656492-172657393) |
| Tfap2c/Limb bud | Centromeric: 172290877 (172290620-172292099)  Telomeric: 172672160 (172670984-172672858) |
| Bmp7/Whole embryo | Centromeric: 172642731 (172642336-172643171)  Telomeric: 172826988 (172826520-172827219) |
| Bmp7/Heart | Centromeric: 172642731 (172642336-172643171)  Telomeric: 172830901 (172830747-172831172) |
| Bmp7/Lateral forebrain | Centromeric: 172630346 (172630152-172631318)  Telomeric: 172850611 (172850060-172851075) |
| Bmp7/Medial forebrain | Centromeric: 172625918 (172625767-172626262)  Telomeric: 172850312 (172849484-172850966) |
| Bmp7/Limb bud | Centromeric: 172636057 (172635953-172636215)  Telomeric: 172845131 (172844296-172845433) |
| Bmp7-3'/Whole embryo | Centromeric: 172630346 (172630152-172630532)  Telomeric: 172836581 (172836259-172837201) |
| Bmp7-3'/Heart | Centromeric: 172630346 (172630152-172630532)  Telomeric: 172835793 (172835079-172836369) |
| TZ/Whole embryo | Centromeric: 172259974 (172259535-172262737)  Telomeric: 172850312 (172848417-172851373) |
| TZ/Heart | Centromeric: 172256655 (172255729-172258747)  Telomeric: 172926570 (172923311-172927540) |

**Table S2.** **Estimated coordinates of the primary interaction domains.**

Coordinates are on chromosome 2, using the NCBI37/mm9 assembly.
