# Supplementary material for: A Discrete Transition Zone Organizes the Topological and Regulatory Autonomy of the Adjacent Tfap2c and Bmp7 Genes
Source: PLoS Genet. 2015 Jan 8;11(1):e1004897. doi: 10.1371/journal.pgen.1004897 (PMC4288730; doi:10.1371/journal.pgen.1004897)
Supplement: S3 Table — Estimated coordinates of the primary interaction domains in rearranged alleles. Coordinates are on chromosome 2, using the NCBI37/mm9 assembly, with those within inverted regions reordered accordingly. (DOCX) [file pgen.1004897.s015.docx]

**Table S3.** **Estimated coordinates of the primary interaction domains in the rearranged alleles.**

Coordinates are on chromosome 2, using the NCBI37/mm9 assembly, with those within inverted regions reordered accordingly.

| **Viewpoint/Allele** | **Coordinates of boundaries (lower-upper estimates)** |
| --- | --- |
| Tfap2c/del1 | Centromeric: 172286001 (172285655-172286673)  Telomeric: 172916160 (172915961-172916359) |
| Tfap2c/INV-M | Centromeric: 172286221 (172285831-172287052)  Telomeric: 172619974 (172619738-172620024) |
| Tfap2c/INV-L2 | Centromeric: 171692638 (171691606-171693086)  Telomeric: 172060348 (172059677-172060695) |
| Bmp7/del1 | Centromeric: 172336479 (172336091-172336669)  Telomeric: 172851883 (172851655-172851992) |
| Bmp7/INV-M | Centromeric: 172649666 (172649537-172650022)  Telomeric: 172830747 (172830332-172830901) |
| Bmp7/INV-L2 | Centromeric: 172290763 (172289936-172291663)  Telomeric: 172919296 (172918274-172920569) |
| Bmp7-3'/INV-M | Centromeric: 172281029 (172280382-172281559)  Telomeric: 172636400 (172635715-172636537) |
| Bmp7-3'/INV-L2 | Centromeric: 170638250 (170635662-170641531)  Telomeric: 171758584 (171756834-171761304) |
| TZ/INV-M | Centromeric: 172261585 (172259596-172263460)  Telomeric: 172916161 (172915542-172917862) |
| TZ/INV-L2 | Centromeric: 170682198 (170680434-170684612)  Telomeric: 172109650 (172106180-172111524) |
