# Supplementary material for: A Discrete Transition Zone Organizes the Topological and Regulatory Autonomy of the Adjacent Tfap2c and Bmp7 Genes
Source: PLoS Genet. 2015 Jan 8;11(1):e1004897. doi: 10.1371/journal.pgen.1004897 (PMC4288730; doi:10.1371/journal.pgen.1004897)
Supplement: S1 Text — Supplemental Materials and Methods. (DOCX) [file pgen.1004897.s016.docx]

**Supplemental Information**

**Supplemental Materials and Methods**

**Generation of the transgenic lines SB-B(3end) and BA0758.**

The targeting construct comprised: the SB8 transposon [79], which carries a *loxP* site and a *LacZ* reporter gene under the control of the human beta-globin minimal promoter inside the transposon cargo; an additional *loxP* site outside of the transposon; and a neomycin resistant gene under the control of the PGK promoter that are flanked by two FRT sequences. The homology arms for the recombination (chr2:172686051-172689701 and chr2:172689702-172694528 (mm9) on the centromeric and telomeric sides, respectively) were cloned through PCR-amplification from the genomic DNA of E14 ES cells with the primers listed in the table below, and then attached to the targeting construct above.

| **Primers used to amplify the homology arms for targeting construct** |
| --- |
| Centromeric side |
| 5’-GGTCGCGGCCGCGTCGACCATTAAACAGATCAGTGCTCACTGG-3’ |
| 5’-CTTTGCGGCCGCTGGGGTCAAGAGAGCTGAACTTTC-3’ |
| Telomeric side |
| 5’-GACTCGAGAGGGTCTTTCAGTCAACATTAAG-3’ |
| 5’-CTGAGTCGACTTTGGAATGGCTCTGCCAGTC-3’ |

The linearized targeting construct was electroporated in E14 ES cells. Drug-resistant colonies were picked and analyzed by PCR-genotyping and Southern blot hybridization using external probes. Positive clones were expanded and injected into donor C57BL/6J blastocyst. Germline-transmission was obtained from one chimera. The FRT-flanked selection cassette was then removed breeding with hACTB-FLPe mice [87], leaving only the transposon and the *loxP* sequence outside of it at the site. The resulting allele is SB-B(3end).

The ES clone BA0758 was obtained from BayGenomics and injected to establish a *Tfap2c*-gene trap line. The correct integration of the gene-trap cassette in *Tfap2c* was verified by PCR.

**Remobilisation of the SB transposon and generation of chromosomal rearrangements.**

The SB-B(3end) mice were mated with animals carrying a transposase Prm1::HSB16-B transgene to generate seed males (carrying both the transposon and the transposase). Seed males were crossed with C57BL/6J females. F1 animals resulting from this cross were genotyped as described before [14] to identify remobilised insertions of the transposon. The genomic position of these insertions was determined by asymmetric PCR [14]. Genomic positions of the insertions used here are given in Table S1. The different genomic deletions, duplications and inversions were produced by *in vivo* genomic engineering [54,18], using the 129S1/Sv-*Hprt^tm1(cre)Mnn^*/J CRE line [80]. The new alleles were identified and verified by PCR genotyping. The primers and schemes to PCR-genotype the transposon lines and the genomic rearrangements are listed in the tables below. The different mouse lines were backcrossed and maintained on C57BL/6J.

Mouse experiments were conducted in accordance with the principles and guidelines in place at European Molecular Biology Laboratory, as defined and overseen by its Institutional Animal Care and Use Committee, in accordance with the European Convention 18/3/1986 and Directives 86/609/EEC and 2010/63/EU.

| **Primers used to genotype mouse lines** | | | |
| --- | --- | --- | --- |
| Primer number | Primer name | Sequence 5'>3' | UCSC mouse genomic coordinate (mm9) |
| 3 | sb_Rir1 | GTTTTGGCAAGTCAGTTAGGACATC | NA |
| 6 | SB_Lir1 | TTTCATCACATTCCCAGTGGGTC | NA |
| 114 | LacZ_5DSrev | TTGAGGGGACGACGACAGTATC | NA |
| 115 | LacZ_3DSup | GGTCGCTACCATTACCAGTTGG | NA |
| 426 | SB-L3 | AAGTAGATGTCCTAACTGACTTGC | NA |
| 428 | SB-R2 | GTGGTGATCCTAACTGACCTAAGAC | NA |
| 1129 | p107 Reverse Primer | CTGCAGGCTAGAAGCAAATG | NA |
| 1608 | Bmp7-SA-out(3´)-r | GATCCACTAGTTCTAGAGCGGC | NA |
| 2413 | SB>Bmp7-gtyp-rev | CCGAGGTTATGGGGATTTTT | Chr2:172690009-172690028 (-) |
| 2414 | SB>Bmp7_5end-gtyp-fwd | GGCTTGCCTATGGCATTAAA | Chr2:172689233-172689252 (+) |
| 2953 | LacZ-bGeo-rev | GTGCTGCAAGGCGATTAAGT | NA |
| 2954 | del(BA0758-Bmp7)-fwd | GGCTTCACCCACATGTCTTT | Chr2:172382145-172382164 (+) |
| 3377 | SB8-199232-R | AGTTATCTTTTGCAACCCAGGA | Chr2:172555788-172555809 (+) |
| 3553 | SB8-202440-R#2 | CTAGGCTCAAGAAAAGCTCAGT | Chr2:172540508-172540529 (-) |
| 3689 | SB8-199232-L | CACCACACTTCATAAACCACAC | Chr2:172556225-172556246 (-) |
| 3782 | Bmp7SB-R#2 | TGGGCTTTTATGGTGTTTCTTA | Chr2:172689721-172689742 (-) |
| 3783 | Bmp7SB-L#2 | GAGGCTTTCTCCAAGGTCAC | Chr2:172689480-172689499 (+) |
| 3784 | SB8-205312-R | GTATCCGAGCTTACCACATGAC | Chr2:167095887-167095908 (+) |
| 3785 | SB8-205312-L | GGTCACTTCACACATGACAAAC | Chr2:167096284-167096305 (-) |
| 3786 | SB8-205918-R | GCTGTCCCTCAATAACAATCAT | Chr2:171656359-171656380 (+) |
| 3787 | SB8-205918-L | TCATCTCCATAGCTGTCTTCCT | Chr2:171656757-171656778 (-) |
| 3788 | SB8-206049-R | CTGCTGTGCCTTCAAGTTTAGA | Chr2:172678600-172678621 (+) |
| 3789 | SB8-206049-L | GTTCTCCCTCCATCTTGTTCTT | Chr2:172678999-172679020 (-) |
| 3790 | SB8-206063-R | TGTGGAGATCTTTTCTCCTAGC | Chr2:168618060-168618081 (-) |
| 3791 | SB8-206063-L | CTGTAAACAACAGAGCCATGAA | Chr2:168617615-168617636 (+) |
| 3792 | SB8-206300-R | GTGAAGTCTTGCTACTGGGAGT | Chr2:172782917-172782938 (+) |
| 3793 | SB8-206300-L | CCCAACCCTTGATTTTTAATTT | Chr2:172783296-172783317 (-) |
| 3794 | SB8-200801-R | AGGATGTCCACATTGTCCTG | Chr2:172745679-172745698 (+) |
| 3795 | SB8-200801-L#2 | CCATGTGAACATTAAAGTCGAA | Chr2:172746103-172746124 (-) |
| 3796 | SB8-202440-L#2 | AACACACCTTGACCAGGACAG | Chr2:172540200-172540220 (+) |
| 3797 | SB8-198819-L | TGGACAAGATCCCTATGTTTTT | Chr2:172690774-172690795 (-) |
| 3798 | SB8-200879-R | GAGCTGGTAACTAGGAGGATGA | Chr2:172667230-172667251 (+) |
| 3799 | SB8-200879-L#2 | ATGCATGCGTATGTGTGTTT | Chr2:172667634-172667653 (-) |

| **Primer combinations used to genotype mouse lines** | | | | |
| --- | --- | --- | --- | --- |
| Line type | Genotype | Primer pair | Size of the product(s) | Remarks |
| Gene-trap line | BA0758 | #2953/2954 | 731 bp |  |
| Transposon lines | SB-B(3end) | #426/3783 | 486 bp | left |
|  |  | #428/2413 | 470 bp | right |
|  | SB-B(up) | #1129/3793 | 577 bp | left |
|  |  | #428/3792 | 366 bp | right |
|  | SB-B(in) | #1129/3795 | 566 bp | left |
|  |  | #428/3794 | 422 bp | right |
|  | SB-A1 | #1129/3796 | 468 bp | left |
|  |  | #428/3553 | 404 bp | right |
|  | SB-A2 | #1129/3689 | 575 bp | left |
|  |  | #428/3377 | 426 bp | right |
|  | SB-198819 | #1129/3797 | 540 bp | left |
|  |  | #428/3376 | 411 bp | right |
|  | SB-200879 | #426/3799 | 352 bp | left |
|  |  | #428/3798 | 314 bp | right |
|  | SB-206049 | #1129/3789 | 588 bp | left |
|  |  | #428/3788 | 383 bp | right |
|  | SB-Sall4 | #426/3791 | 352 bp | left |
|  |  | #428/3790 | 357 bp | right |
|  | SB-205918 | #1129/3787 | 488 bp | left |
|  |  | #428/3786 | 410 bp | right |
|  | SB-205312 | #1129/3785 | 556 bp | left |
|  |  | #428/3784 | 405 bp | right |
| Deletion lines | del1 | #3553/3782/3796 | 521 bp (del)  330 bp (wt) |  |
|  | del1+LacZ | #1129/3796 | 468 bp | SB-A1_left |
|  |  | #428/2413 | 470 bp | SB-B(3end)_right |
|  | del2+LacZ | #2953/2954 | 442 bp |  |
|  | del3+LacZ | #426/3791 | 352 bp | SB-Sall4_left |
|  |  | #428/2413 | 470 bp | SB-B(3end)_right |
| Inversion lines | INV-L1 | #3782/3784/3785 | 689 bp (inv)  419 bp (wt) |  |
|  | INV-L2 | #3782/3786/3787 | 628 bp (inv)  459 bp (wt) |  |
|  | INV-M | #3377/3689/3782 | 628 bp (inv)  459 bp (wt) |  |
|  | INV-Bmp7 | #3782/3792/3793 | 630 bp (inv)  401 bp (wt) |  |
| Universal | SB-LacZ | #3/114 | 622 bp |  |
|  |  | #6/115 | 568 bp |  |
|  | fixed loxP at SB-B(3end) | #1608/2414 | 494 bp |  |
|  | deletion | #3/2414/3782 | 383 bp (del)  510bp (wt) |  |
|  | inversion | #1129/2413/3783 | 375 bp (inv)  549 bp (wt) |  |

**LacZ staining.**

Mouse embryos collected at stages E10.5, E11.5 and E12.5 were fixed in 4% PFA/PBS on ice for 30, 30 and 40 minutes, respectively. After three washes in PBS, embryos were put in staining solution (0.01% Sodium Deoxycholate, 0.02% NP-40, 2 mM MgCl_2_, 270 l NP-4spermidine, 3.3 mg/ml K_3_Fe(CN)_6_, 4.2 mg/ml K_4_Fe(CN)_6_ e(CN_2_O and 0.8 mg/ml X-gal in PBS, pH 7.4) and then incubated overnight at 37°C in a dark humid chamber. Embryos were washed with PBS and stored at 4°C. PCR-genotyping of embryos was performed on genomic DNA extracted from extra-embryonic membranes.

**Whole-mount *in situ* hybridization**

*Tfap2c* and *Bmp7* probes were generated from templates generated by PCR from mouse cDNA and cloned in pGEM-T Easy (Promega) (primers listed in the table below). DIG-UTP-labeled antisense RNAs (Roche) were produced by *in vitro* transcription of the linearized temples using SP6 RNA polymerase (Roche).

Mouse embryos used for *in situ* hybridization were dissected and fixed in 4% PFA/PBS overnight. The extra-embryonic membranes were used for genotyping by PCR. Whole-mount *in situ* hybridization was carried out following standard protocol.

| **Primers used to clone the probe templates** |
| --- |
| *Tfap2c* |
| 5’-ACCAGCCGCCTCCTTACTTCCC-3’ |
| 5’-ACGTGGGCAGCTTTCCGTCTCC-3’ |
| *Bmp7* |
| 5’-AACGAGGTGCACTCCAGC-3’ |
| 5’-GTCTTGGAGCGATTCTGGC-3’ |

**Isolation of RNA from embryonic tissues - RT-qPCR**

For all experiments, we compared stage-matched embryos (maximum 2 somite-difference) from the same litter, obtained by breeding heterozygous males and females. The tissues dissected out from embryos were frozen in liquid nitrogen and stored at -80°C. Genotyping was performed by PCR on genomic DNA extracted from extra-embryonic membranes. Total RNA was extracted from the frozen tissues using RNeasy kit (QIAGEN) with DNaseI treatment on columns. cDNA was synthesized with the ProtoScript II First Strand cDNA Synthesis Kit (New England Biolabs) and random hexamer primers.

The quantitative PCR was performed using StepOne Real-Time PCR System with SYBR green reagent (Applied Biosystems). The primers used are listed in the table below. For each gene, we measured the amplification efficiency of the primers and used these values to calculate the relative expression level from the Ct values obtained by the PCR. To quantify *Tfap2c* and *Bmp7* expression level, we used cDNA plasmid clones as standards. *Gapdh* expression was used as a standard to normalize expression level for each sample. For all conditions, we used at least three biological replicates, and calculated the mean and the standard deviation of the replicates. Statistical significance was determined by a two-tailed Student’s t-test.

| **Primers used for RT-qPCR** |
| --- |
| *Tfap2c* |
| 5’-GGGAGGTGTGCTCAGAAGAG-3’ |
| 5’-GACGTGAGGAGAGTGACGTG-3’ |
| *Bmp7* |
| 5’-GAAAACAGCAGCAGTGACCA-3’ |
| 5’-AGGTCTCGGAAGCTGACGTA-3’ |
| *Gapdh* |
| 5’-CTCCCACTCTTCCACCTTCG-3’ |
| 5’-CCACCACCCTGTTGCTGTAG-3’ |
| *Ptgis* |
| 5’-ATGCCTTGGAGTTTGGGAGAG-3’ |
| 5’-AGGAGAACAGTGACGTATCTGC-3’ |
| *Dok5* |
| 5’-CGAATGGTGCAAAGTTCTCCAG-3’ |
| 5’-ACCCCGGTAGCCAATAAATCAG-3’ |

***In vivo* enhancer assay**

We cloned the FB1 enhancer (chr2:172551998-172555000, mm9) from a BAC clone (RP24-103P2, CHORI) by recombineering in EL250 cells [88]. The linear shuttle vector used for recombineering was amplified by PCR so as to add corresponding homology sequences flanking FB1 to its extremities (GTGGCTCAGCAGGCAAATGAGCTTGCCATCAAGACTGATGGCCTGGGTTCTCTAGACGCGGGGAGAGGCGGTTTGCGTAT and CAGCAGGGCACACCTTTGATCCCAGCACCACTTCAGAGGCAGAGGCAGACCTCGAGCTGGCCGTCGTTTTACAACGTCGT). This FB1 fragment was subcloned upstream of a LacZ enhancer-reporter construct incorporated in a lentiviral backbone[81]. Proviruses with this FB1-ßlac transgene were produced in HEK293 cells [81]. The virus was concentrated with a Centricon Plus 70 centrifugal Filter Devices (Millipore UFC710008), and micro-injected under the *zona pellucida* of one-cell embryos. Injected embryos were kept in culture in KSOM medium up to the blastocyst stage and reimplanted into pseudo-pregnant foster mothers. They were recovered at stage E11.5, stained for LacZ activity and genotyped.

**3C assay**

To prepare the 3C library we dissected out the heart and the lateral and medial forebrains from E11.5 C57BL/6J embryos as indicated on the figure below. Taking the LacZ staining as a reference, we estimated that FB1 and *Tfap2c* were active in roughly ~ 80 % of the medial forebrain and ~ 70% of the lateral forebrain; 50% of the cardiac cells showed LacZ staining in a SB-B(3end) embryo (figure below). We dissociated the cells by pipetting gently and passed them through Falcon cell strainers with 70-μm pores. We fixed the cells in 12 ml of 2% PFA/PBS at room temperature for 10 min. PFA was quenched by addition of 1.5 ml of 1 M glycine. Cells were washed once in PBS, frozen in liquid nitrogen and stored at -80°C. Samples from three litters were collected independently, pooled and processed as described in [82]. Cells were lysed in lysis buffer (50 mM Tris (pH7.5), 150 mM NaCl, 5 mM EDTA, 0.5% NP-40, 1% Triton X-100, 1x complete proteinase inhibitors (Roche); 1 ml) at 4°C for 10 minutes. Nuclei were lysed in 0.3% SDS for 1 hour, followed by an additional 1 hour-incubation with 2.5% Triton X-100. Cross-linked chromatin was digested overnight at 37°C by *NlaIII*. After heat-inactivation of the restriction enzyme, digested crosslinked chromatin was self-ligated with T4 DNA ligase at 16°C for overnight. The cross-links were then reversed and genomic DNA purified with phenol-chloroform extraction and ethanol precipitation.

To quantify the ligation products, we conducted qPCR with TaqMan probes. The primers and probes, and their combinations used in this assay are listed in the tables below. To quantify the relative ligation frequency of the different regions, we made standard libraries from three BAC clones covering the region (RP24-176A22, RP24-103P2 and RP23-96P16), which were mixed in a equal molar ratio, cut with *NlaIII*, ligated and purified. The regions overlapped by two different BAC clones were excluded from the analysis. To normalize the ligation efficiency among different libraries, we used one locus from a different chromosome (chr8:95215868-95220938) that has seemingly no regulatory activity in the tissues considered here. We designed primers and probe pairs to capture proximity-dependent ligation of two fragments 5 kb apart from each other. The ligation frequency of this control was normalized by using a standard ligation product obtained for the BAC clone RP23-406E4. All quantifications by Taqman qPCR were done with four technical replicates, and their mean and the standard deviation were calculated and plotted.


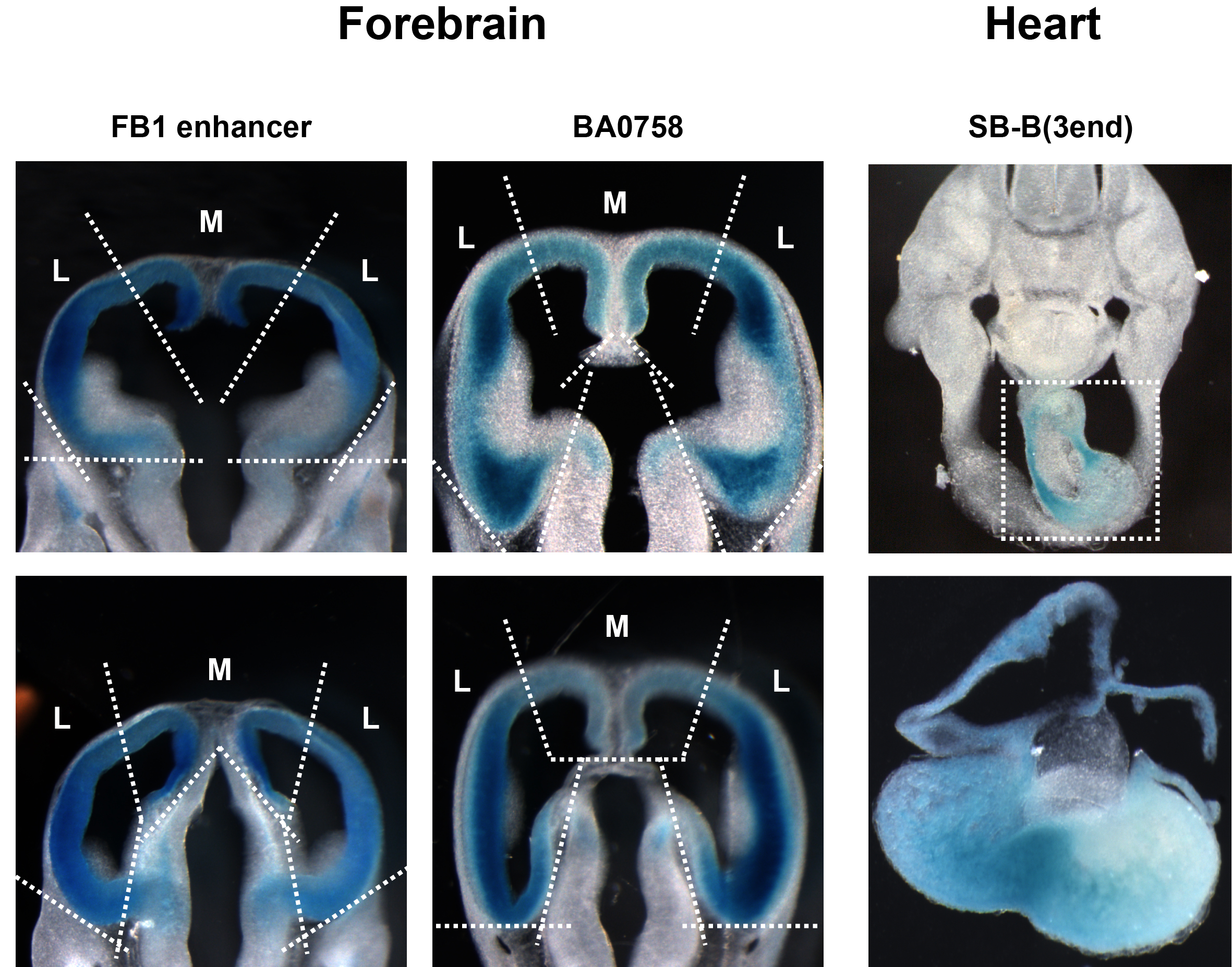


Close-up view of forebrain (150µm section) and heart illustrating the proportion of LacZ positive/expressing cells for the different tissues, as well as the landmarks used to dissect the lateral (L) and medial (M) forebrain.

| **Primers used for 3C-qPCR assay** | | | |
| --- | --- | --- | --- |
| Number | Sequence 5' > 3' | UCSC mouse genomic coordinate (mm9) | Remarks |
| 4537 | TTTGCAGAAGGAAGCAAGCCTCG | Chr2:172553373-172553395 (+) | FB1 probe, 5'-FAM 3'-BHQ |
| 4539 | ACTGGAGCAAATGGAGTGTTGGG | Chr2:172766450-172766472 (-) | Bmp7 probe, 5'-FAM 3'-BHQ |
| 4540 | ATATCTCAGGGCGACCTCCCTGTA | Chr2:172374201-172374224 (+) | Tfap2c probe, 5'-FAM 3'-BHQ |
| 4541 | GTCATAACAACTTCAGAGAAAGGAAAG | Chr2:172553396-172553422 (-) | FB1 primer used with 4537 |
| 4542 | CGTGTTAAAGCCTGCTCAGA | Chr2:172688945-172688964 (+) |  |
| 4543 | CTTGGACCTGGCTCATCTTT | Chr2:172766416-172766435 (+) | Bmp7 primer used with 4539 |
| 4544 | CGTGGAATGAGGTGCTGTAA | Chr2:172374239-172374258 (-) | Tfap2c primer used with 4540 |
| 4546 | CACCTCGCAATCCTCTTCATAC | Chr2:172375364-172375385 (-) |  |
| 4547 | CACGACCCGAGGTCACTTG | Chr2:172765649-172765667 (+) |  |
| 4548 | GCCAGAACTGAGTAAAGGACAG | Chr2:172765742-172765763 (-) |  |
| 4552 | CATCTCTAGAATTCAGTGGTGTCC | Chr2:172784971-172784994 (+) |  |
| 4553 | TGTGGGCTATAAGACCGTAGT | Chr2:172717885-172717905 (+) |  |
| 4554 | CAGTCTGTGACTTTGGACCTG | Chr2:172717013-172717033 (-) |  |
| 4555 | TGTCTGGGAAGACAGGAAGA | Chr2:172356107-172356126 (+) |  |
| 4556 | TAGTGTCAGAATGCCACGTAAA | Chr2:172328309-172328330 (+) |  |
| 4557 | CAGCCTTCCAGTTTCCTCTT | Chr2:172327929-172327948 (-) |  |
| 4559 | AACTTCAGAGAAAGGAAAGCGA | Chr2:172553393-172553414 (-) |  |
| 4561 | CTGACACTCACCAGAGAAACC | Chr2:172551111-172551131 (-) |  |
| 4562 | AGAGCGTTATATCCTCCAGTCC | Chr2:172488322-172488343 (+) |  |
| 4563 | CCTCGGACCTGAGACCTATTT | Chr2:172486987-172487007 (-) |  |
| 4564 | TTTCCCAGCTCACAGACAAG | Chr2:172521017-172521036 (+) |  |
| 4565 | CAGCAGACCTGCCTCATAAT | Chr2:172521091-172521110 (-) |  |
| 4568 | CTAAGTTGGCAGAGTCAGAGTG | Chr2:172688370-172688391 (-) |  |
| 4569 | CCATTGTGTGTCTCTCCTTGT | Chr2:172664586-172664606 (+) |  |
| 4571 | GGCTTAAGATGCATAGGTGTCC | Chr2:172646536-172646557 (+) |  |
| 4572 | CTTCATCAACCGTGGCTTCATA | Chr2:172645787-172645808 (-) |  |
| 4573 | CCAATCTGGCTAGGAAGTGAAG | Chr2:172565558-172565579 (+) |  |
| 4575 | CAATTTGACAGGGTCTGGACT | Chr2:172431061-172431081 (+) |  |
| 5005 | CAGGTATGGAGTCCCAAATCA | Chr8:95220900-95220920 (+) | Chr8 primer fwd |
| 5006 | AAACTCTGCCAGGAGGAAG | Chr8:95215951-95215969 (-) | Chr8 primer rev |
| 5007 | ATAAACCCTTCCCAGCGTCCTGG | Chr8:95215915-95215937 (+) | Chr8 probe, 5'-FAM 3'-BHQ |

| **Primers combinations for 3C-qPCR assay** | |
| --- | --- |
| Fragment pairs | Pairs of probes and primers |
| Tfap2c-1 | #4540/4544/4556 |
| Tfap2c-2 | #4540/4544/4555 |
| Tfap2c-3 | #4540/4544/4575 |
| Tfap2c-4 | #4540/4544/4563 |
| Tfap2c-5 | #4540/4544/4565 |
| Tfap2c-6 | #4540/4544/4561 |
| Tfap2c-7 | #4540/4544/4559 |
| Tfap2c-8 | #4540/4544/4573 |
| Tfap2c-9 | #4540/4544/4572 |
| Tfap2c-10 | #4540/4544/4554 |
| Tfap2c-11 | #4540/4544/4548 |
| Tfap2c-12 | #4540/4544/4552 |
| Bmp7-1 | #4539/4543/4555 |
| Bmp7-2 | #4539/4543/4546 |
| Bmp7-3 | #4539/4543/4575 |
| Bmp7-4 | #4539/4543/4564 |
| Bmp7-5 | #4539/4543/4559 |
| Bmp7-6 | #4539/4543/4573 |
| Bmp7-7 | #4539/4543/4571 |
| Bmp7-8 | #4539/4543/4569 |
| Bmp7-9 | #4539/4543/4568 |
| Bmp7-10 | #4539/4543/4542 |
| Bmp7-11 | #4539/4543/4553 |
| Bmp7-12 | #4539/4543/4552 |
| FB1-1 | #4537/4541/4557 |
| FB1-2 | #4537/4541/4555 |
| FB1-3 | #4537/4541/4546 |
| FB1-4 | #4537/4541/4575 |
| FB1-5 | #4537/4541/4562 |
| FB1-6 | #4537/4541/4565 |
| FB1-7 | #4537/4541/4573 |
| FB1-8 | #4537/4541/4572 |
| FB1-9 | #4537/4541/4553 |
| FB1-10 | #4537/4541/4547 |
| FB1-11 | #4537/4541/4552 |
| Standard (chr8) | #5005/5006/5007 |

**4C library preparation, sequencing and the data analysis**

The 3C libraries were first prepared as described above for the E11.5 whole embryos and E12.5 limb buds, except that the single cell nuclei were prepared by passing the fixed and lysed cells through needles for several times. *NlaIII-*digested and re-ligated DNA from the 3C libraries was digested by a second enzyme (*DpnII*) and then self-ligated. After the ethanol precipitation, the eluted DNA was purified twice with QIAquick PCR Purification Kit (QIAGEN). The concentration of the DNA was measured by NanoDrop. An inverse PCR reaction was performed with the primers listed in the table below, using 3.2 µg of the libraries as templates except for the INV-M whole embryo sample, for which we used 1.8 µg. The reading primers included a 3-6 nucleotide-long tag sequence, specific to each library, between the Illumina read adaptor and the annealing sequence. The PCR products were purified with the High Pure PCR Product Purification Kit (Roche), and subsequently with QIAquick PCR Purification Kit (QIAGEN). The libraries were mixed and sequenced on a HiSeq 2000 (Illumina).

Sequencing reads FASTQ files were demultiplexed using the first 8 bases of the barcode and viewpoint primer sequences, allowing no mismatch. The minimum Hamming distance between these octamer sequences was 2. In the same step, we trimmed the primer sequence, keeping the restriction enzyme cutting site at the read starts. We aligned the demultiplexed 4C libraries to the mm9 reference genome using Bowtie version 1.0.0 [83]. We generated a “4C reference genome” by cutting the mm9 mouse genome *in silico* for *NlaIII* (recogination site = CATG). To filter the reads of the 4C libraries, we mapped the aligned reads to this 4C reference genome. Only reads starting exactly at a restriction fragment end with the correct orientation were assigned to the corresponding fragment end. We summed up the counts for both ends of a fragment into one count value per fragment.

To normalize for the different library sizes, we divided the counts by the total number of counts on the viewpoint chromosome (chr2) for each library and multiplied these values by 1,000,000 (“RPM normalization”). For visualization, we smoothed the counts over adjacent fragments, using a window size of 11 fragments. The smoothened 4C read counts were shown on the normal reference genome, or for the inversion alleles on the corresponding reconstructed ones. For each inversion, we inverted the underlying coordinates of the reference genome, and represented the normalized smoothened 4C read-counts on these corrected versions. To avoid border effects, we did not include the two fragments that contained the inversion breakpoints. For the samples with genomic deletions, we used a similar approach.

To estimate the primary interaction domain for each viewpoint, we adapted a well-established segmentation approach initially implemented for the analysis of micro-array data [59]. This approach fits piecewise constant functions to the input data. The resulting change points of these fits define the segment boundaries. We used this algorithm on the 4C signal of each viewpoint, segmenting the signal into 3 segments (primary interaction domain, left and right region outside the primary interaction domain). For each viewpoint we removed all fragments that contained 0 counts across all individual experiment and applied the algorithm to each experiment individually.

To compare the read distributions for the viewpoints in the transition zone, we calculated the counts in different windows using RPM normalized data. A 10-kb window around the viewpoint was excluded from the analysis. The first window was the inverted region on chromosome 2 with the breakpoints at the genomic positions 172556090 and 172689701 (INV-M). Adjacent windows to the inverted region were 400 kb to the centromeric and telomeric sides of the chromosome. In each window, we calculated the number of counts. Using the total number of counts in these 3 windows, the fractions shown in Figure S8 were calculated.

To quantify asymmetries in the interaction frequencies of viewpoints we calculated cumulative count distributions to each side of the viewpoint. For this, we used RPM normalized counts to be able to compare between viewpoints. Because the fragments directly adjacent to the viewpoint had a high number of counts, they strongly influenced the cumulative distribution. To reduce this strong confounding signal, we removed the counts of fragments from the analysis whose genomic distance to the viewpoint was less than 10 kb.

| **Primers used to prepare 4C libraries** | | |
| --- | --- | --- |
| Sample library | Viewpoint | Sequence |
| INV-M (whole embryo) | A | AATGATACGGCGACCACCGAACACTCTTTCCCTACACGACGCTCTTCCGATCTATGGCATTCCTATGATGCTGTCCATG |
| WT (whole embryo) | A | AATGATACGGCGACCACCGAACACTCTTTCCCTACACGACGCTCTTCCGATCTCATCATTCCTATGATGCTGTCCATG |
| INV-L2 (whole embryo) | A | AATGATACGGCGACCACCGAACACTCTTTCCCTACACGACGCTCTTCCGATCTGCTGCATTCCTATGATGCTGTCCATG |
| WT (heart) | A | AATGATACGGCGACCACCGAACACTCTTTCCCTACACGACGCTCTTCCGATCTTCTGAGCATTCCTATGATGCTGTCCATG |
| INV-M (whole embryo) | Tfap2c | AATGATACGGCGACCACCGAACACTCTTTCCCTACACGACGCTCTTCCGATCTAGCTAGTCAACAACCCTCCTCCCATG |
| WT (whole embryo) | Tfap2c | AATGATACGGCGACCACCGAACACTCTTTCCCTACACGACGCTCTTCCGATCTATCGTGTCAACAACCCTCCTCCCATG |
| INV-L2 (whole embryo) | Tfap2c | AATGATACGGCGACCACCGAACACTCTTTCCCTACACGACGCTCTTCCGATCTCAGGTCAACAACCCTCCTCCCATG |
| WT (heart) | Tfap2c | AATGATACGGCGACCACCGAACACTCTTTCCCTACACGACGCTCTTCCGATCTCCGTATCAACAACCCTCCTCCCATG |
| WT (lateral forebrain) | Tfap2c | AATGATACGGCGACCACCGAACACTCTTTCCCTACACGACGCTCTTCCGATCTGCTATCAACAACCCTCCTCCCATG |
| WT (medial forebrain) | Tfap2c | AATGATACGGCGACCACCGAACACTCTTTCCCTACACGACGCTCTTCCGATCTGGAATCAACAACCCTCCTCCCATG |
| WT (limb bud) | Tfap2c | AATGATACGGCGACCACCGAACACTCTTTCCCTACACGACGCTCTTCCGATCTTCGTCAACAACCCTCCTCCCATG |
| del1 (whole embryo) | Tfap2c | AATGATACGGCGACCACCGAACACTCTTTCCCTACACGACGCTCTTCCGATCTTGCCGTCAACAACCCTCCTCCCATG |
| INV-M (whole embryo) | B | AATGATACGGCGACCACCGAACACTCTTTCCCTACACGACGCTCTTCCGATCTATTAACAATGTCCAACCTAGCATG |
| WT (whole embryo) | B | AATGATACGGCGACCACCGAACACTCTTTCCCTACACGACGCTCTTCCGATCTGGCCAACAATGTCCAACCTAGCATG |
| INV-L2 (whole embryo) | B | AATGATACGGCGACCACCGAACACTCTTTCCCTACACGACGCTCTTCCGATCTGTATAGAACAATGTCCAACCTAGCATG |
| WT (heart) | B | AATGATACGGCGACCACCGAACACTCTTTCCCTACACGACGCTCTTCCGATCTTGGAGAACAATGTCCAACCTAGCATG |
| INV-M (whole embryo) | Bmp7 | AATGATACGGCGACCACCGAACACTCTTTCCCTACACGACGCTCTTCCGATCTAGCGTCCATTTGCTCCAGTCATG |
| WT (whole embryo) | Bmp7 | AATGATACGGCGACCACCGAACACTCTTTCCCTACACGACGCTCTTCCGATCTATAGTCCATTTGCTCCAGTCATG |
| INV-L2 (whole embryo) | Bmp7 | AATGATACGGCGACCACCGAACACTCTTTCCCTACACGACGCTCTTCCGATCTCACTGTCCATTTGCTCCAGTCATG |
| WT (heart) | Bmp7 | AATGATACGGCGACCACCGAACACTCTTTCCCTACACGACGCTCTTCCGATCTCCACTCCATTTGCTCCAGTCATG |
| WT (lateral forebrain) | Bmp7 | AATGATACGGCGACCACCGAACACTCTTTCCCTACACGACGCTCTTCCGATCTGCCTCCATTTGCTCCAGTCATG |
| WT (medial forebrain) | Bmp7 | AATGATACGGCGACCACCGAACACTCTTTCCCTACACGACGCTCTTCCGATCTTATATCCATTTGCTCCAGTCATG |
| del1 (whole embryo) | Bmp7 | AATGATACGGCGACCACCGAACACTCTTTCCCTACACGACGCTCTTCCGATCTTCCGTCCATTTGCTCCAGTCATG |
| WT (limb bud) | Bmp7 | AATGATACGGCGACCACCGAACACTCTTTCCCTACACGACGCTCTTCCGATCTTGATCCATTTGCTCCAGTCATG |
| common to all | A | CAAGCAGAAGACGGCATACGAGCTTTAGAGCTTGGTTCAGA |
| common to all | Tfap2c | CAAGCAGAAGACGGCATACGAGATAGGCTCACAACGAAGTC |
| common to all | B | CAAGCAGAAGACGGCATACGAATGGAGCTGTGGACTAGATC |
| common to all | Bmp7 | CAAGCAGAAGACGGCATACGAGGGGAAGTCAGTCCTCCC |

**SUPPORTING INFORMATION ADDITIONAL REFERENCES**

87. Rodríguez CI, Buchholz F, Galloway J, Sequerra R, Kasper J, et al. (2000) High-efficiency deleter mice show that FLPe is an alternative to Cre-loxP. Nat Genet 25: 139–140.

88. Lee EC, Yu D, Martinez de Velasco J, Tessarollo L, Swing DA, et al. (2001) A highly efficient Escherichia coli-based chromosome engineering system adapted for recombinogenic targeting and subcloning of BAC DNA. Genomics 73: 56–65.
